# The TFPI-2 Derived Peptide EDC34 Improves Outcome of Gram-Negative Sepsis

**DOI:** 10.1371/journal.ppat.1003803

**Published:** 2013-12-05

**Authors:** Praveen Papareddy, Martina Kalle, Ole E. Sørensen, Martin Malmsten, Matthias Mörgelin, Artur Schmidtchen

**Affiliations:** 1 Division of Dermatology and Venereology, Department of Clinical Sciences, Lund University, Biomedical Center, Lund, Sweden; 2 Division of Infection Medicine, Department of Clinical Sciences, Lund University, Biomedical Center, Lund, Sweden; 3 Department of Pharmacy, Uppsala University, Uppsala, Sweden; 4 Lee Kong Chian School of Medicine, Nanyang Technological University, Singapore, Singapore; Yale University School of Medicine, United States of America

## Abstract

Sepsis is characterized by a dysregulated host-pathogen response, leading to high cytokine levels, excessive coagulation and failure to eradicate invasive bacteria. Novel therapeutic strategies that address crucial pathogenetic steps during infection are urgently needed. Here, we describe novel bioactive roles and therapeutic anti-infective potential of the peptide EDC34, derived from the C-terminus of tissue factor pathway inhibitor-2 (TFPI-2). This peptide exerted direct bactericidal effects and boosted activation of the classical complement pathway including formation of antimicrobial C3a, but inhibited bacteria-induced activation of the contact system. Correspondingly, in mouse models of severe *Escherichia coli* and *Pseudomonas aeruginosa* infection, treatment with EDC34 reduced bacterial levels and lung damage. In combination with the antibiotic ceftazidime, the peptide significantly prolonged survival and reduced mortality in mice. The peptide's boosting effect on bacterial clearance paired with its inhibiting effect on excessive coagulation makes it a promising therapeutic candidate for invasive Gram-negative infections.

## Author Summary

Bacterial infections, especially sepsis, are worldwide a major cause of morbidity and mortality. Sepsis is characterized by an excessive and uncontrolled immune and coagulation response caused by bacteria and bacterial products, which eventually leads to multiple organ failure. Despite supportive treatments and administration of antibiotics, the incidence of sepsis is rising. Development of antibiotic resistance among bacteria, and the inability of antibiotics to target dysregulated host responses during severe infections and sepsis, motivates the search for novel anti-infective treatment modalities. Here, we describe a therapeutic potential of the peptide EDC34, derived from the C-terminus of tissue factor pathway inhibitor-2 (TFPI-2). The peptide's boosting effect on bacterial clearance paired with its inhibiting effect on excessive coagulation makes it a promising therapeutic candidate for invasive Gram-negative infections.

## Introduction

Sepsis is a major cause of death in the western world, with mortality ranging between 30 and 70% [1]. The disease is characterized by an excessive and dysregulated immune and coagulation response to microbial infections, leading to capillary leakage, lung damage, and finally multiple organ failure [2], [3], [4]. Several clinical trials targeting coagulation as well as pro-inflammatory responses have been conducted, including administration of activated protein C [5], [6], antibodies against TNF-α [7], [8], [9], interleukin-1 receptor antagonists [10], [11], interleukin-6 antagonists [12], anti-endotoxin antibodies [12], PAF receptor antagonists [13], antithrombin III [14], [15], [16] and other agents [17], [18], [19], [20]. Even though animal experiments showed promising results, all drug candidates tested so far have failed to show clinical efficiency. Consequently, today's treatment for sepsis is largely based on antibiotics in combination with supportive measures, illustrating the need for new therapeutic approaches.

Antimicrobial peptides constitute one group of compounds, which have recently attracted attention as new anti-infectives. Due to their preferential interactions with prokaryotic and fungal membranes, these peptides provide a rapid and broad-spectrum response towards both Gram-negative and Gram-positive bacteria, as well as fungi [21], [22], [23], [24]. Antimicrobial peptides also mediate diverse immunomodulatory roles, including anti-inflammatory effects as well as stimulation of chemotaxis, chemokine production, wound healing and angiogenesis [25], [26], [27], motivating the term host defense peptides (HDP). The molecular diversity of HDPs has provided a wide range of structures of potential interest for the anti-infective field. For example, immunomodulatory peptides such as IDRs (Innate Defense Regulator) selectively protect against bacterial infection by chemokine induction and neutrophil recruitment, while reducing pro-inflammatory cytokine responses [26], [27]. Further, the lactoferrin-derived peptide hLF1-11 differentiates monocytes, which enhances clearance of pathogens, a feature currently utilized in the development of therapies for infections in immunosuppressed patients [28]. Additionally, C-terminal peptides of human thrombin, exerting anti-inflammatory, anti-coagulative and antimicrobial effects, are effective in ameliorating LPS-induced shock and *Pseudomonas* sepsis in experimental settings in animals [29], [30], [31], further exemplifying that endogenous HDPs may have therapeutic potential.

The present work is based on the finding that the highly positively charged C-terminus of tissue factor pathway inhibitor-2 (TFPI-2) encodes for antimicrobial activity [32]. Previous data demonstrated that C-terminal TFPI-2 fragments are released in human wounds, and can be generated by neutrophil elastase *in vitro*. A direct antimicrobial effect of a prototypic 34 amino acids long C-terminal TFPI-2 peptide, EDC34 was furthermore demonstrated *in vitro* [32]. In this work, utilizing various *in vitro* and *in vivo* models aimed at characterizing effects on complement, coagulation, and bacterial clearance, we demonstrate a therapeutic potential of the peptide for the treatment of Gram-negative infections.

## Results

### The C-terminal TFPI-2 peptide EDC34 is antimicrobial and its effect enhanced in plasma and blood

**Figure 1:**
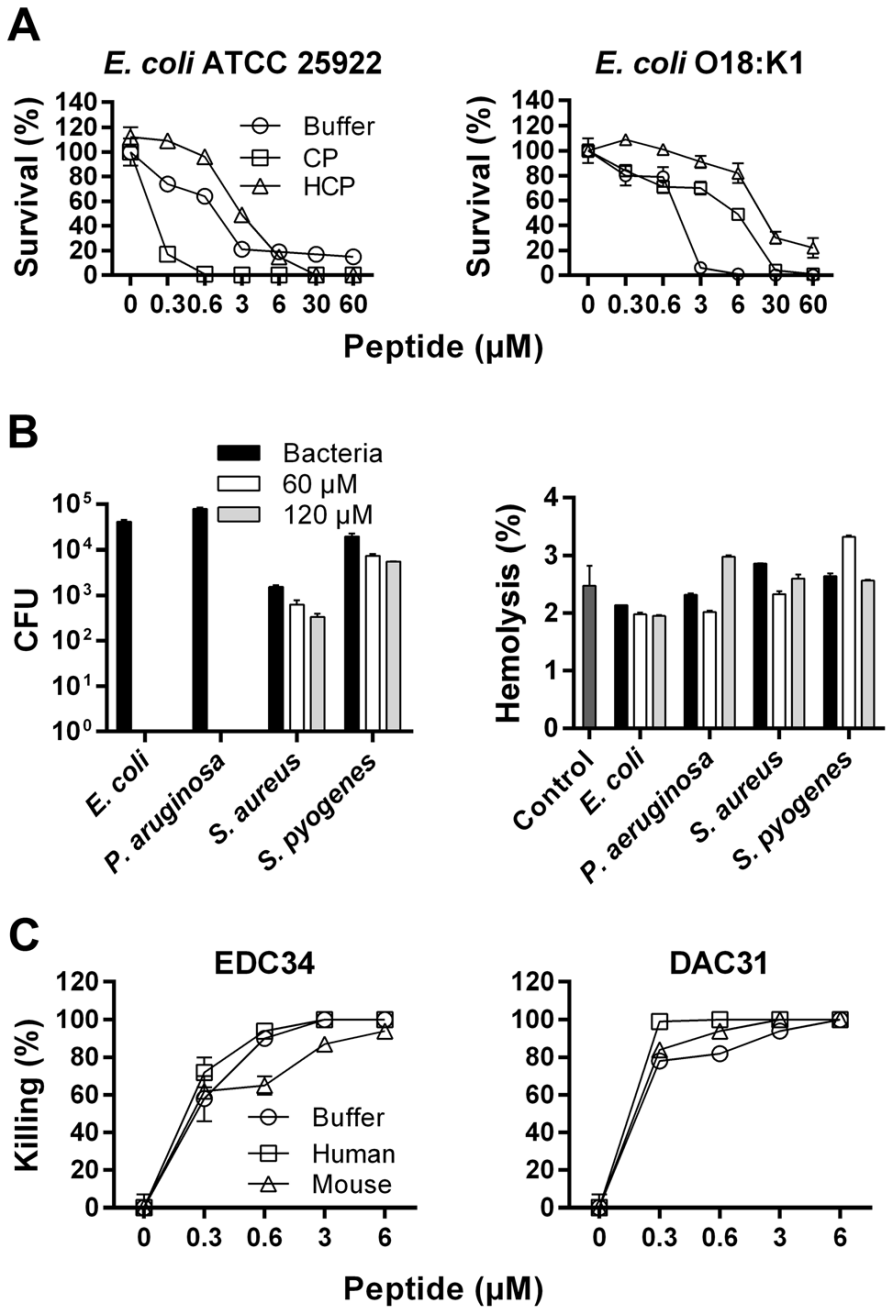
Antimicrobial activities of EDC34. (**A**) Antibacterial effects of EDC34 at the indicated concentrations were studied in viable count assays, using the bacteria *E. coli* ATCC 25922 (complement sensitive) and *E. coli* O18:K1 (complement-resistant). Analyses were performed in 10 mM Tris, 0.15 M NaCl, pH 7.4 (Buffer) or buffer containing 20% citrate plasma (CP) or heat-inactivated citrate plasma (HCP). Mean with SD is shown (n = 3). (**B**) Antimicrobial and hemolytic effects of EDC34 against the indicated bacteria in whole blood (n = 3, mean±SEM is presented). (**C**) Antimicrobial effects of EDC34 and DAC31, a mouse derived TFPI-2 C-terminal peptide against *E. coli* in human or mouse citrate plasma (n = 3, mean±SEM is presented).

Initial experiments utilizing *E. coli* and *P. aeruginosa* isolates demonstrated that EDC34 displayed significant antibacterial activity in physiological buffer, and enhanced activity in the presence of human plasma (**Figure 1A** **and Figure S1A and B**). Heat inactivation of human plasma abolished this potentiating effect. A control peptide (DAA14) derived from the N-terminal part of TFPI-2 did not show any antimicrobial effects in buffer or plasma (data not shown). In contrast, the cathelicidin LL-37 was partially inhibited by the addition of native as well as heat-inactivated plasma (**Figure S1A and B**), which was compatible with previous observations showing that the peptide's activity is compromised in presence of plasma due to protein binding [33]. Thus, these data suggested that the antibacterial effect of EDC34 is dependent on *additional* bactericidal systems in normal plasma, such as complement. As described for LL-37, it was observed that EDC34 showed reduced antibacterial activity in heat-inactivated plasma, particularly against the *E. coli* strains 25922, 49.1 and 47.1 when compared to buffer (**Figure 1A** **and Figure S1A**), possibly related to scavenging effects of plasma proteins. In human whole blood, EDC34 demonstrated potent antimicrobial effects against various *E. coli* and *P. aeruginosa* isolates at doses as low as 0.3 µM, except for *P. aeruginosa* PA01, which was killed at 3 µM (**Figure S1C and D**). Notably, 10–100 times higher doses were required for LL-37 mediated killing (**Figure S1C and D**). Kinetic studies in the presence of plasma demonstrated that the bacterial killing mediated via EDC34 (3 µM) occurred within 10–20 min for *E. coli*, and 40–60 min for *P. aeruginosa* (**Figure S2A and B**). Contrary to results for a complement-susceptible *E. coli* strain (**Figure 1A** **, left panel**), no EDC34-mediated enhancement of bacterial killing was observed for the *E. coli* O18:K1 strain, previously shown to be resistant to complement-mediated lysis [34] (**Figure 1A** **, right panel**). Next, antibacterial and hemolytic effects of EDC34 were simultaneously analyzed in human blood. Consistent with the complement-dependent action of EDC34, this peptide was active against Gram-negative *E. coli* and *P. aeruginosa* in whole blood, while exhibiting little or no effects against Gram-positive *S. aureus* and *S. pyogenes* AP1 (**Figure 1B** **, left panel**). No significant increase in hemolysis was observed at peptide doses up to 120 µM (**Figure 1B** **, right panel**). Next, the fact that the human EDC34 sequence differs from the related murine sequence prompted us to compare the effects of human EDC34 with those of the related peptide of murine origin (DAC31), derived from a corresponding C-terminal region of murine TFPI-2 (**Table S1**). Both peptides exerted similar antimicrobial effects in buffer as well as in human and mouse plasma (**Figure 1C**). Taken together, the data imply that bacterial killing by EDC34 in plasma and blood is enhanced by the presence of an intact complement system.

### EDC34 induces complement activation at bacterial surfaces

**Figure 2:**
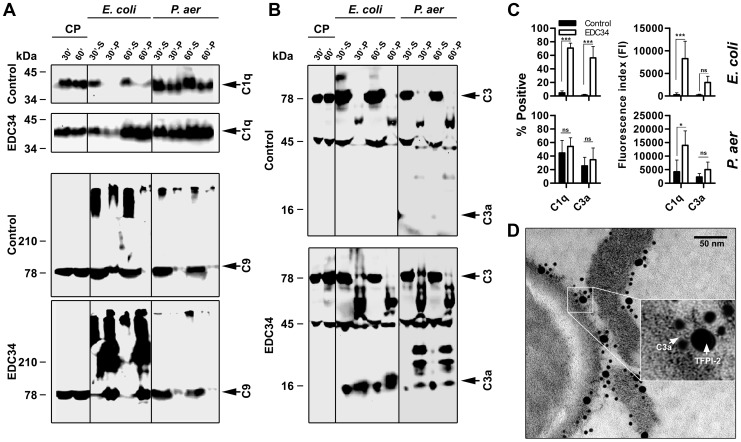
EDC34 enhances the binding of complement proteins to bacteria. (**A**) *E. coli* ATCC 25922 and *P. aeruginosa* PA01 bacteria were incubated 30–60 min with citrate plasma alone (Control) or supplemented with EDC34 (at 3 µM) at 37°C. The bacterial cells and supernatants were collected and proteins were detected by immunoblotting with antibodies recognizing C1q or C5b-9. CP, citrate plasma; S, supernatant or unbound bacteria; P, pellet or material bound to bacterial cells. (**B**), as in figure 2A, but antibodies against C3a were used. (**C**) Quantification of deposition of complement components on bacteria by flow cytometry, *left panel*, comparison of the mean proportion of bacteria positive for C1q/C3a binding in citrate plasma and in plasma supplemented with EDC34. *Right panel*, comparative degree of C1q and C3a binding to *E. coli* and *P. aeruginosa* strains, expressed as means of the fluorescence index (*FI*; proportion of bacteria positive for C1q/C3a multiplied by the mean intensity of C1q/C3a binding). (n = 4, mean±SEM is presented; Two-Way ANOVA Bonferroni's Multiple Comparison Test). (**D**) TFPI-2 and C3a binding to bacteria in fibrin slough collected from an infected chronic wound were visualized by using gold-labeled antibodies of different sizes, specific for the C-terminus of TFPI-2 (20 nm) and C3a (10 nm), respectively. Insert shows a higher magnification.

In order to study the complement boosting effect of EDC34 further, western blot and FACS analyses were used. EDC34 enhanced binding of C1q to *E. coli* (**Figure 2A**), and increased the formation of the membrane attack complex (MAC), as evidenced by increased binding of C9 and related high molecular weight compounds (**Figure 2A**). A significant generation of C3a was also observed after addition of EDC34 (**Figure 2B–D** **and Figure S3**). After subjecting *P. aeruginosa* to plasma, in contrast to the results with *E. coli* above, an activation of complement by the bacteria *per se* was observed (**Figure 2A–D** **and Figure S3**). Nevertheless, quantification of C1q after addition of EDC34 detected an increase of this molecule in association with *P. aeruginosa* (**Figure 2C** **and Figure S2**). Additionally, fragments corresponding to bacterial-bound C3a, as well as peptides of higher molecular weight containing the C-terminal epitope of C3a, were detected, particularly after addition of EDC34 (**Figure 2B–D**). Electron microscopy studies on fibrin slough from a patient with a chronic wound infected by *P. aeruginosa* were furthermore performed to explore a possible co-localization between the C-terminal TFPI-2 region and C3a *in vivo*. Using immunogold-labeled antibodies against the C-terminal part of TFPI-2 and against C3a, the evaluation of 50 bacterial profiles indeed showed that ∼70% of C-terminal TFPI-2 peptide epitopes were closely associated with C3a (**Figure 2D**). Taken together, these results indicate that EDC34 promotes complement activation in response to *E. coli* and *P. aeruginosa*.

### EDC34 inhibits the intrinsic pathway of coagulation

**Figure 3:**
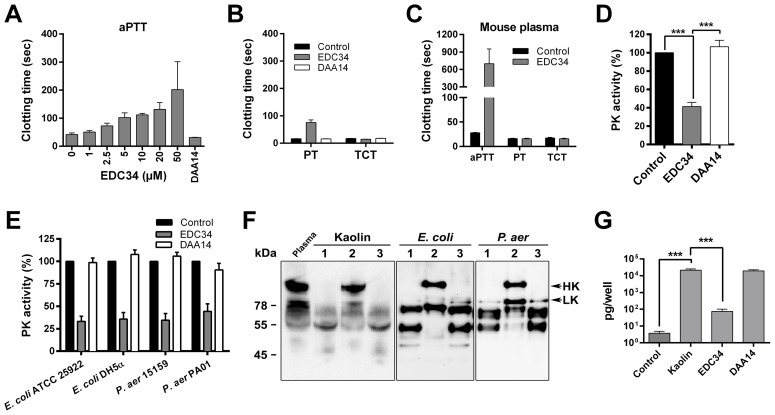
EDC34 inhibits contact activation at negatively charged surfaces. (**A**) Effect of EDC34 on the contact system was assessed by measuring the aPTT in human citrate plasma. The control peptide DAA14 was used at 50 µM. (**B**) Measurement of effects of EDC34 (50 µM) and DAA14 (50 µM) on the extrinsic (PT) and common pathway (TCT) of coagulation. (**C**) Measurement of aPTT, PT and TCT clotting times of mouse citrate plasma in absence (Control) or presence of 50 µM EDC34. (**D**) Plasma kallikrein activity induced by negatively charged kaolin in human citrate plasma in absence (Control) or presence of EDC34 (50 µM) or DAA14 (50 µM), was determined by a chromogenic substrate assay. Data are presented as mean±SEM relative to control without peptides (n = 4; One-Way ANOVA Bonferroni's Multiple Comparison Test). (**E**) Plasma kallikrein activity in human citrate plasma induced by the indicated bacteria in absence (Control) or presence of the peptides EDC34 (50 µM) or DAA14 (50 µM) was measured by a chromogenic substrate assay. Data are presented as mean±SEM relative to control without peptides (n = 5). (**F**) Western blot analysis of HK and HK degradation products: 1, No peptide; 2, EDC34; 3, DAA14. (**G**) Bradykinin release in human citrate plasma incubated with kaolin in absence (Control) or presence of EDC34 (50 µM) or DAA14 (50 µM) (n = 3, mean±SEM is presented; One-Way ANOVA Bonferroni's Multiple Comparison Test).

TFPI-2 inhibits TF-VII-mediated coagulation and affects factor XIa and plasma kallikrein [35], [36], effects which are thought to be mediated by the Kunitz-type domains of TFPI-2 [37]. However, no evidence has so far been presented that the C-terminal region of TFPI-2 may influence coagulation. Clotting assays using human citrate plasma revealed that EDC34 interfered with the intrinsic pathway of coagulation, as illustrated by a dose-dependent prolongation of the activated partial thromboplastin time (aPTT) (**Figure 3A**). At 50 µM, the increase in aPTT was about 4-fold in human plasma (**Figure 3A**) and 20-fold in mouse plasma (**Figure 3C**). In contrast, the other parts of the coagulation system, such as the extrinsic pathway of coagulation, monitored by measuring the prothrombin time (PT), and thrombin-induced fibrin-network formation (thrombin clotting time; TCT), were not affected or to a lower extent at doses of 20–50 µM (**Figure 3B and C**, and data not shown). The activation of the intrinsic system takes place at negatively charged surfaces, such as kaolin or bacteria, and involves activation of FXII, which then leads to the activation of plasma kallikrein (PK) and FXI [38]. Analysis of PK activity at the surface of kaolin showed that EDC34, but not the control peptide from the N-terminal region of TFPI-2 (DAA14), was able to block the PK activity (**Figure 3D**). In order to determine whether EDC34 inhibits PK activity also on bacterial surfaces, *P. aeruginosa* and *E. coli* bacteria, sensitive to the peptide in plasma and blood, were chosen for further studies. Bacteria were pre-incubated with EDC34, followed by incubation with plasma, and the effect of EDC34 and the control peptide recorded by measuring the PK activity. The results showed that only EDC34 was able to inhibit PK activation (**Figure 3E**). Since high molecular weight kininogen (HMWK) is a substrate for PK [38], western blots were utilized to assess possible degradation of HMWK in plasma. Compatible with the results of the PK assay, EDC34 blocked the degradation of HMWK (**Figure 3F**). PK-cleaved HMWK releases bradykinin (BK), a potent pro-inflammatory mediator [38], and corresponding to the above, EDC34 also significantly inhibited the generation of bradykinin (**Figure 3G**). The N-terminal TFPI-2 control peptide DAA14 neither inhibited PK activation nor bradykinin generation (**Figure 3D–G**). These results show that EDC34 mainly inhibits activation of the intrinsic pathway of coagulation, leading to reduced HMWK degradation and bradykinin release.

### EDC34 modulates coagulation *in vivo* but does not inhibit LPS-induced cytokine responses *in vitro* or *in vivo*

**Figure 4:**
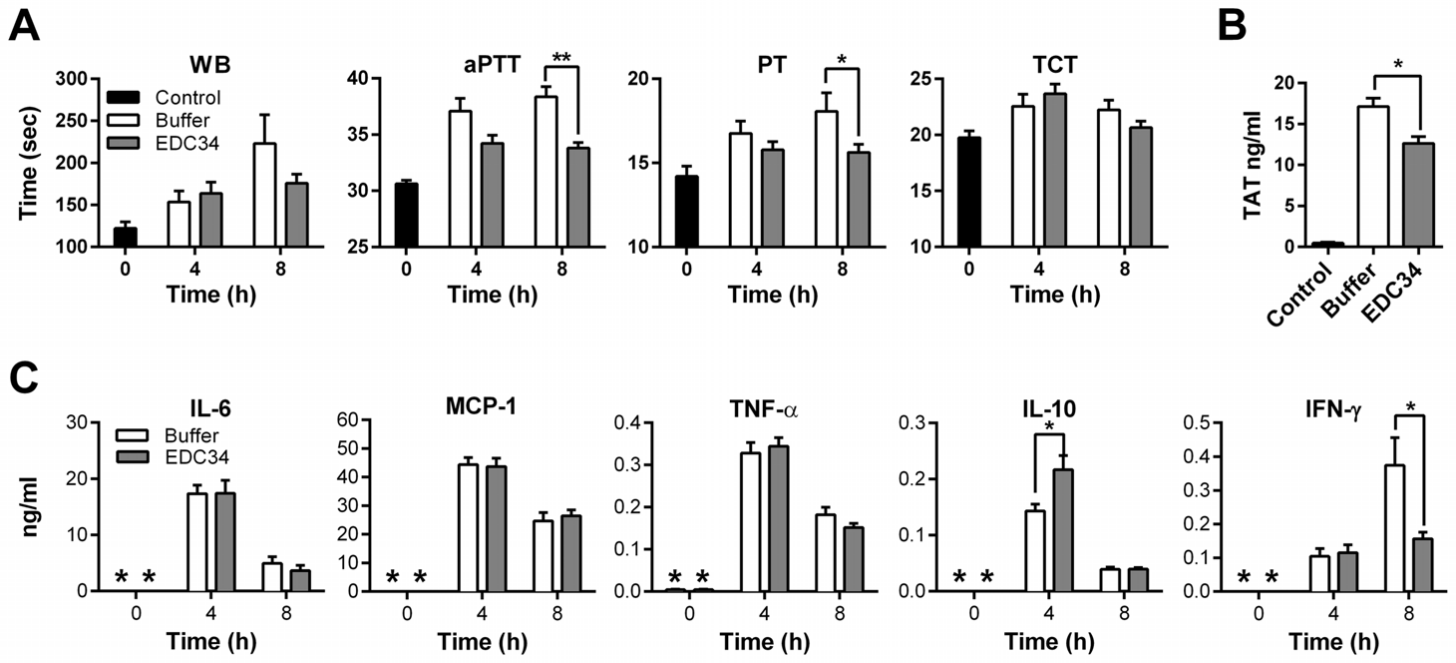
EDC34 is anti-coagulative, but not anti-inflammatory during LPS-shock. C57BL/6 mice were injected i. p. with 5 mg/kg *E. coli* LPS O111:B4 and treated after a period of 30 min with buffer (Buffer) or EDC34 (0.5 mg, i. p.). Mice were sacrificed 4 and 8 h after LPS injection and (**A**) clotting times of whole blood (WB) or in citrate plasma (aPTT, PT, TCT), (n = 9/group, mean±SEM is presented; Two-Way ANOVA Bonferroni's Multiple Comparison Test), (**B**) Thrombin-antithrombin complexes (TAT) (n = 8/group, mean±SEM is presented; One-Way ANOVA Bonferroni's Multiple Comparison Test) and (**C**) cytokines were determined (in C, * indicates below detection level) (Control; n = 8, Buffer and EDC34; n = 9/group, mean±SEM is presented; Two-Way ANOVA Bonferroni's Multiple Comparison Test).

To assess possible anti-coagulative effects *in vivo*, 12 mg/kg of LPS was injected into the mice followed by intraperitoneal administration (i. p.) of 0.5 mg of EDC34 after 30 min. Measurements of whole blood clotting, aPTT, PT, and TCT at 4 h and 8 h after LPS challenge clearly demonstrated an anti-coagulant effect of EDC34 in this model (**Figure 4A**). This effect was confirmed by determining thrombin-antithrombin complexes (TAT) in mouse plasma 8 h after LPS injection. TAT complexes were reduced in EDC34-treated animals (**Figure 4B**). Previous studies have demonstrated that EDC34 binds LPS similarly to LL-37, compatible with its direct antibacterial effects on bacteria [32]. To test whether LPS-binding confers any anti-inflammatory effects to EDC34, mouse macrophages were stimulated with LPS in presence or absence of EDC34. GKY25, a thrombin-derived LPS-binding and anti-inflammatory peptide, was used as positive control [29], [31]. In contrast to GKY25, which blocked the LPS response at 1–5 µM, EDC34 did not exert any endotoxin-blocking effects, even at significantly higher concentrations (**Figure S4**). Previously published work showed that GKY25 (at 0.5 mg) significantly improved survival as well as inhibited cytokine responses in a mouse model of endotoxin shock [29], [31]. In contrast, and corresponding to the absence of anti-inflammatory effects *in vitro*, the same dose (0.5 mg) of EDC34, when administrated i. p. 30 minutes after endotoxin exposure, was not able to block IL-6, MCP-1, and TNF-α responses, although it affected the production of the anti-inflammatory IL-10 at an early time point (**Figure 4C**), and reduced IFN-γ after 8 h. Thus, these results show that EDC34 largely modulates coagulation during LPS-induced shock *in vivo*.

### EDC34 is antimicrobial and anti-coagulative in an *Escherichia coli* animal infection model

**Figure 5:**
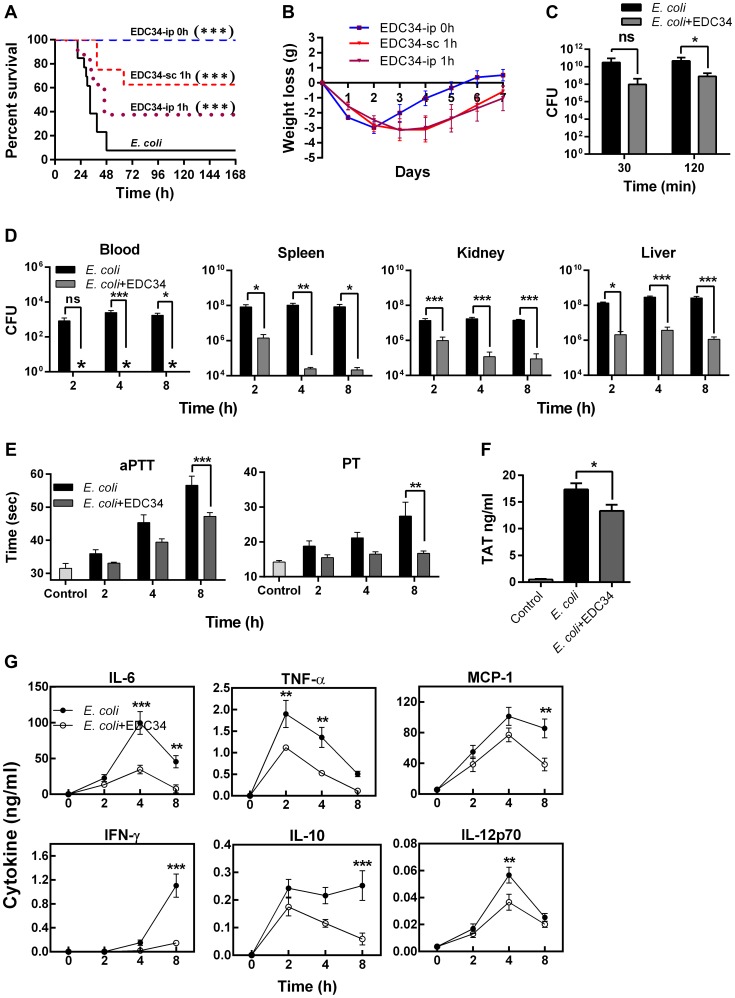
EDC34 is antimicrobial and anti-coagulative in an *E. coli* infection model *in vivo*. BALB/c mice were injected (i. p.) with *E. coli* DH5-α (2×10^8^ cfu) and treated with EDC34 (0.5 mg) as indicated in **A** and **B**. (**A**) Survival is presented (*E. coli* infection; n = 13, treatment with EDC34 i.p., or sc; n = 8, Log-rank Mantel-Cox test) and (**B**) weight was determined daily. As only one animal survived in the untreated group (after day 2), the weight curve for untreated mice is not shown (EDC34-ip. 0 h vs. EDC34-sc. 1 h (***); EDC34-ip. 0 h vs. EDC34-ip. 1 h (*); and EDC34-sc. 1 h vs. EDC34-ip. 1 h (ns), mean±SEM is presented; Two-Way ANOVA Bonferroni's Multiple Comparison Test). (**C–G**) BALB/c mice were injected (i. p.) with *E. coli* DH5-α (1.5×10^8^ cfu) and immediately treated with EDC34 (0.5 mg, i. p.). The following parameters were determined: (**C**) Cfu in peritoneal wash fluid (n = 15/group, mean±SEM is presented; Two-Way ANOVA Bonferroni's Multiple Comparison Test), (**D**) cfu in blood and indicated organs (n = 9/group, mean±SEM is presented; Two-Way ANOVA Bonferroni's Multiple Comparison Test), (**E**) aPTT and PT in citrate plasma (n = 9/group, mean±SEM is presented; Two-Way ANOVA Bonferroni's Multiple Comparison Test), (**F**) TAT complexes (8 h post-infection, n = 8/group, mean±SEM is presented; One-Way ANOVA Bonferroni's Multiple Comparison Test), and (**G**) cytokines (Control, 0 h; n = 7, *E. coli* and EDC34 treatment; n = 9/group, mean±SEM is presented; Two-Way ANOVA Bonferroni's Multiple Comparison Test).

Taken together, the above data provided a rationale for using the human peptide EDC34 in murine Gram-negative infection models. Hence, mice were infected i. p. with *E. coli*, followed by i) immediate i. p. treatment with EDC34, ii) delayed i. p. treatment with the peptide given after 1 h, or iii) s. c. treatment after 1 h, in order to separate peptide and bacteria, and minimize direct peptide effects during i. p. administration. Notably, in these models, immediate as well as delayed EDC34 treatment administered i. p. or s. c. yielded significant improvements in survival when compared to controls (**Figure 5A**). Although all treated mice displayed a significant weight loss, it was observed that the i. p. treated animals recovered faster (**Figure 5B**). The i. p. model using immediate treatment was selected in order to evaluate peptide effects in greater detail. Mice were infected i. p. with *E. coli*, followed by treatment with EDC34. Two hours post-infection, a significant reduction of bacterial levels in the peritoneal cavity was detected only in peptide-treated animals (**Figure 5C**). To assess the importance of complement activation for the observed antibacterial effects of EDC34 *in vivo*, C3a levels were measured in peritoneal fluid and plasma (**Figure S5A and B**). In peritoneal fluid, treatment with EDC34 indeed yielded increased C3a levels, in particular after 2 hours post-infection (**Figure S5A**). These results were compatible with the observed reduction of bacterial counts in the peritoneum of these EDC34-treated animals (**Figure 5C**). Concomitantly, C3a levels in plasma of peptide-treated animals were reduced, reflecting the local boosting effect of EDC34 at the site of infection leading to overall reduced bacterial levels and hence, relatively less complement activation systemically (**Figure S5B**). The importance of an intact complement system was further demonstrated in experiments employing pretreatment with cobra venom factor (CVF), used in order to deplete animals of complement factors [39] before infection and peptide treatment. In this infection model, the antibacterial effect of EDC34 was significantly compromised in CVF-treated mice when compared to control mice (**Figure S5C**). Taken together, these experiments are compatible with the previous *in vitro* experiments presented in Figure 2, and firmly demonstrate that EDC34 mediates its antibacterial effect *in vivo* by boosting of complement activation. Having shown this, a second set of *in vivo* experiments with *E. coli*, aimed at studying peptide effects on bacterial dissemination, cytokines, and coagulation parameters after 2, 4, and 8 h post-infection were performed. The results showed that treatment with EDC34 yielded significantly lower bacterial levels in the spleen, kidney, liver and blood when compared to the controls (**Figure 5D**). Notably, infected mice showed a reduced clotting capacity and exhibited prolonged aPTT and PT times, along with an increase in TAT complexes in their plasma. Treatment with EDC34 however, resulted in an improved clotting function, as evidenced by reduced coagulation times (**Figure 5E**) and TAT complex formation (**Figure 5F**). These data implied that the peptide, by enhancing bacterial clearance and blocking bacteria-induced coagulation, reduced the excessive consumption of coagulation factors in this animal model, thus improving hemostasis function. It is of note that time-dependent cytokine responses were detected also in EDC34 treated animals, although at lower levels when compared with non-treated infected animals. (**Figure 5G**). However, this reduction of cytokine responses was not unexpected, since the bacterial levels were reduced 10–100 times by EDC34 (**Figure 5D**). Hence, the lower, *but retained* cytokine response was compatible with the noted absence of significant anti-inflammatory effects of EDC34 in the LPS-shock model above (**Figure 4C**).

### Treatment with EDC34 improves outcome in experimental *P. aeruginosa* sepsis

**Figure 6:**
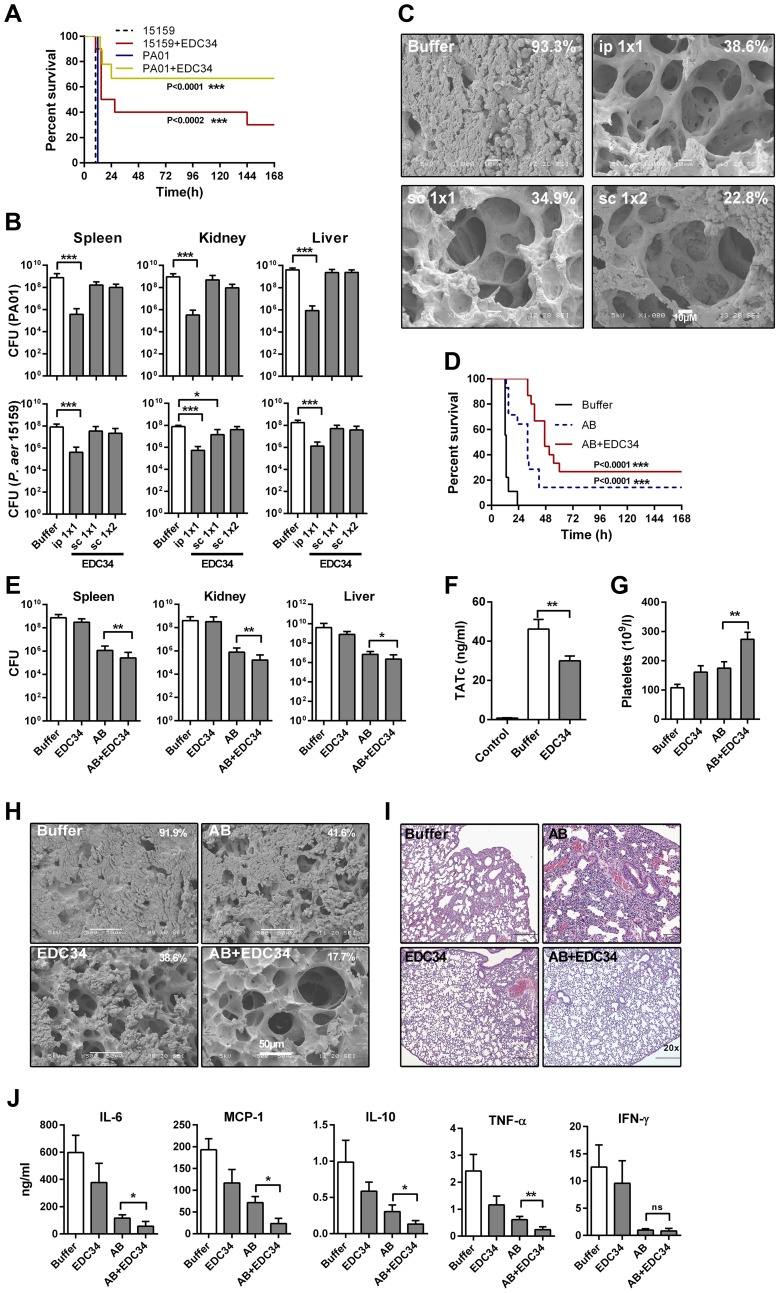
Effects of EDC34 in a *P. aeruginosa* infection model. (**A**) C57BL/6 mice were injected (i. p.) with *P. aeruginosa* PAO1 or 15159 followed by i. p. injection of EDC34 (0.5 mg) or buffer only (n = 9/group; log-rank Mantel-Cox test). (**B**) C57BL/6 mice were injected i. p. with *P. aeruginosa* PA01 or 15159 bacteria and treated immediately after inoculation with 0.5 mg of EDC34 injected i. p. or s. c. at 1 h (1×1) or 1 and 7 h (1×2) after bacterial infection. (For *P. aeruginosa* PA01 infection model, control; n = 18, i. p. 1×1; n = 9, s. c. 1×1; n = 13, s. c. 1×2; n = 8; One-Way ANOVA Bonferroni's Multiple Comparison Test) (For *P. aeruginosa* 15159 infection model n = 8/group, mean±SEM is presented). Cfu were determined 12 h post-infection. (**C**) Animal lungs of *P. aeruginosa* PA01 infected animals (see B for details) were analyzed by scanning electron microscopy 12 h post-infection (n = 3, and a representative lung section is shown). Percentage of fibrin deposition is indicated. (**D**) C57BL/6 mice were injected i. p. with *P. aeruginosa* 15159, followed by s. c. treatment after 1.5 h with buffer (Buffer), ceftazidime (AB) (300 mg/kg) or EDC34 (0.5 mg) or a combination of ceftazidime (300 mg/kg) and EDC34 (0.5 mg). The injections were repeated 4.5 h after bacterial injection. Survival was determined for 7 days (n = 10/group, P = 0.0002; log-rank Mantel-Cox test). (**E–J**) C57BL/6 mice were injected i. p. with *P. aeruginosa* 15159, followed by s. c. treatment after 1.5 h with ceftazidime (AB) (300 mg/kg) or EDC34 (0.5 mg) or a combination of ceftazidime (300 mg/kg) and EDC34 (0.5 mg). The injections were repeated 4.5 h post-infection. (**E**) Bacterial cfu were determined 10 h post-infection (Buffer; n = 10, ceftazidime (AB); n = 13, combination (AB+EDC34); n = 12). (**F**) Thrombin-antithrombin complexes in plasma were analyzed from a separate *in vivo* experiment. Mean±SEM is presented and compared by Mann-Whitney U-test (Control; n = 3, Buffer; n = 22, EDC34; n = 18). (**G**) Platelets were analyzed in blood from animals of the above experiment. (**H**) From the above experiment, lungs of the animals were collected 10 h post-infection and analyzed by SEM (n = 3, and a representative lung section is shown). Percentage of fibrin deposition is indicated. (**I**) A representative lung section, stained with hematoxylin and eosin, taken 10 h post-infection is shown. For scoring and statistics of a larger set of samples, see Supplementary Fig. S6. (**J**) Cytokines were analyzed in blood from the above experiment.

To further investigate the functions of EDC34 in a mouse model of *Pseudomonas*-induced sepsis, two strains of *P. aeruginosa*, PA01 and 15159 (the latter a clinical isolate), were used. The bacteria were injected i. p., and the peptide was immediately administered either by i. p. injection (1×1) or s. c., either 1 h (1×1) or 1 and 7 h (1×2) after bacterial injection. EDC34 yielded significant improvements in survival after immediate treatment (**Figure 6A**), but failed to improve survival after delayed treatment (not shown). Corresponding to the survival results, a reduced number of bacteria was observed in spleen, kidney, and liver compared to control mice, for both strains, after immediate treatment with EDC34 (**Figure 6B**). Delayed treatment s. c. yielded no significant reduction of bacterial levels. However, and compatible with the peptide's anti-coagulative actions *in vitro* and *in vivo*, scanning electron microscopy (SEM) analyses of the lungs from mice infected with *P. aeruginosa* PAO1 showed that EDC34, irrespective of administration route, reduced fibrin deposition and pulmonary leakage of proteins and red blood cells (**Figure 6C**).

### Effects of EDC34 in combination with ceftazidime during *Pseudomonas aeruginosa* infection

Although EDC34 lowered initial bacterial levels after i. p. administration, the peptide did not completely eradicate bacteria in the above infection models, particularly noted for the clinical isolate. The activation of the intrinsic coagulation system by bacteria underlies the excessive coagulation and bradykinin-induced vascular leakage, and is therefore of interest to target during *P. aeruginosa* infection. Since antibiotics do not inhibit these bacterial effects on coagulation, we decided to explore the multiple effects of EDC34 in combination with ceftazidime, an antibiotic often used in Gram-negative infections. In an infection model mimicking a potential clinical situation, bacteria were injected i. p., followed by treatment 90 min and 4.5 h after bacterial challenge with either ceftazidime (300 mg/kg) or EDC34 (0.5 mg s. c.) alone, or the antibiotic in combination with EDC34 (doses as above). In contrast to animals treated with ceftazidime alone, the combination treatment significantly improved survival of the animals (**Figure 6D**). In contrast, the peptide alone did not increase survival in this model (not shown). Further, bacterial levels were monitored in the spleen, kidney, and liver. Ceftazidime-treated animals had 100-fold less bacterial levels, which were further slightly reduced after addition of EDC34 (**Figure 6E**), while EDC34 alone did not significantly reduce bacteria. Nevertheless, in spite of similar bacterial levels (**Figure 6E**), the generation of TAT complexes was reduced in EDC34-treated mice compared to controls (**Figure 6F**), and thrombocyte levels were increased after peptide treatment. This increase in thrombocytes was also noted in combination with the antibiotic, indicating a reduced activation of the coagulation system due to peptide treatment (**Figure 6G**). This observation was supported by reduced fibrin deposition and pulmonary leakage of protein and red blood cells in the lungs of animals treated with EDC34 alone, or in combination with ceftazidime as judged by SEM (**Figure 6H**) and confirmed by histochemistry and scoring of lung damage (**Figure 6I** and **Figure S6**). It was noted that animals treated with ceftazidime alone showed lung damage changes, involving reduction of alveolar space, increased cell infiltration and wall thickness, and formation of thrombi, similar to those observed in infected untreated animals (**Figure 6I** and **Figure S6**). EDC34 did not significantly reduce cytokine levels when given alone. Nevertheless, cytokine levels were reduced after treatment with the antibiotic as well as with the combination with EDC34 (**Figure 6J**). EDC34 at a total dose of 2 mg did not significantly affect coagulation (aPTT, PT, TCT), thrombocytes, and cytokines after 12 h when administrated into healthy mice (**Figure S7**).

## Discussion

The development of novel anti-infective treatments has been largely addressing the bacteria itself, or the subsequent dysregulated host response, while efforts to control the dysregulated host response have so far failed. Therefore, there is a need for new therapies that address new targets in the complex interplay between microbes and the host. HDPs with multiple roles, such as those here defined for the TFPI-2-derived EDC34, targeting both bacteria and coagulative mechanisms, should therefore be of therapeutic interest. The main objective in this work was to clarify bioactive effects of EDC34 *in vitro*, and to what extent these can be utilized in anti-infective therapy *in vivo*. Challenging in this perspective was the fact that the *in vitro* effects, as observed in isolated experimental systems, were not independent of each other *in vivo*. For example, reduced bacterial levels could also be linked to concomitant reductions in pro-inflammatory cytokines *in vivo*. In such cases, an attempt was made to reduce complexity, and to study isolated factors or events, such as the peptide's anti-inflammatory effects *in vitro* in relation to LPS-stimulation of macrophages, or *in vivo*, in relation to endotoxin shock. In other cases, we highlight unique and peptide-dependent effects, such as abolished fibrin deposition irrespective of bacterial load and antibiotic usage in the animal models, clearly linking the anti-coagulative effects *in vitro* including blocking of bacteria-induced kallikrein activation and bradykinin release, to those observed *in vivo*. Furthermore, interference by EDC34 with the coagulation system *in vivo* was demonstrated not only by reduced fibrin deposition but also evidenced by reduced TAT levels, along with increased thrombocyte levels. This is of importance, since sepsis- and coagulation-related acute lung injury is considered to be a critical feature compromising the clinical outcome during sepsis [40], [41], [42]. In response to LPS challenge, the coagulation cascade is activated, leading to an excessive activation of the coagulation system, followed by *consumption* of coagulation factors in the blood resulting in prolonged clotting times [43]. In line with this, LPS-injected mice showed a reduced clotting capacity and exhibited prolonged aPTT and PT times in their plasma (**Figure 4**). Treatment with EDC34 however, resulted in a partially normalized clotting function, as evidenced by reduced coagulation times (**Figure 4**). Hence, these data showed that the peptide, by blocking coagulation as shown in Figure 3, *reduced* the excessive consumption of coagulation factors in this animal model. Also of importance, was the observation that no signs of bleeding or prolongation of coagulation was noted in animals treated with EDC34 only (**Figure S7A and B**). This should be of value, since other agents tested in the clinic, such as activated protein C, mainly affect the extrinsic or primary pathway of coagulation, and thus, may increase the risk of bleeding complications [5], [6]. As mentioned above, EDC34 did not present any significant anti-endotoxin effects *in vitro* or *in vivo*, in spite of its binding to LPS [32]. Such absence of direct anti-inflammatory effects may be advantageous, particularly in situations where an anti-coagulative action is of importance, while maintaining a normal LPS immune response.

The complement system is crucial for bacterial clearance, indicating that strategies based on manipulating complement activation and thus bacterial clearance, may be of therapeutic interest. It is therefore notable that EDC34 boosts complement activation rapidly *ex vivo* in relation to the Gram-negative *E. coli* and *P. aeruginosa*. Thus, *E. coli* did not significantly induce activation of the classical complement pathway in absence of EDC34 in plasma *in vitro*, and the boosting effects were dependent on EDC34-binding to the bacteria. Although EDC34 was active against Gram-positive *S. aureus* in low salt buffer conditions [32], the results in human blood, where EDC34 was particularly active against *E. coli* and *P. aeruginosa*, further underscores the importance of the peptide's dependence of an active complement system for its actions in the *in vivo* models. Notably, EDC34-mediated formation of C3a, an anaphylatoxin exerting antimicrobial effects [44], was increased not only *in vitro*, but also locally at the site of infection in the animal *E. coli* infection model. In this context, it was interesting to note that although C3a was *increased* intraperitoneally, the overall levels of C3a in blood of the animals were *reduced* after peptide treatment. At a first sight, this may appear paradoxical, however, it is not unexpected when considering the significant reduction of bacteria systemically in peptide-treated animals. Also relevant in this context is that analyses of levels of C3 in *E. coli* infected animals showed that the total levels of this complement factor were unaffected after treatment i. p. with EDC34 (not shown), indicating that the modulation takes place locally, and that the total levels of C3 may buffer a potential local, initial consumption of the protein in this particular animal model. Furthermore, animals with a compromised complement function due to CVF treatment [39] did not exhibit a boosting of *E. coli* clearance by EDC34 *in vivo*. These results were compatible with the *in vitro* studies using heat-inactivated (hence complement-inactivated) plasma (**Figure 2**), elegantly demonstrating the importance of an intact complement system for the action of EDC34 *in vitro* and *in vivo*.

From a biological perspective, it remains to be investigated whether TFPI-2 adds to host defense *in vivo*. Although these studies are currently initiated and TFPI-2^−/−^ mice have been generated and are currently validated and characterized, this work is clearly beyond the scope of this present work aimed at utilizing the TFPI-2 peptide as a therapeutic molecule. Nevertheless, it is notable that emerging evidence suggests an involvement of TFPI-2 in host defense. Thus, *in vitro*, stimulation of human endothelial cells with inflammatory mediators such as LPS, and TNF-α significantly increases TFPI-2 expression [45]. Analogously, *in vivo* in a murine model, TFPI-2 expression is dramatically upregulated in the liver and in the lungs during LPS stimulation [46], [47]. These data, together with previous findings on release of C-terminal fragments by neutrophil elastase and their presence in human wounds [32], and as shown herein, particularly in association with bacteria (**Figure 2D**), suggest that TFPI-2 fragments may exert physiological roles during infection.

From a clinical perspective infections with Gram-negative bacteria such as *E. coli* and *P. aeruginosa* contribute to morbidity and mortality during abdominal surgery, peritoneal dialysis, burn wounds or in immunocompromized patients [48], [49], [50], [51]. An initial phase of effective bacterial clearance is crucial for patient outcome, and therefore, approaches based on substitution with EDC34 in combination with antibiotics may be of therapeutic interest. In order to verify such possibilities, experimental models are crucial, and it is notable that EDC34 exerted similar antibacterial effects in mouse and in human plasma, which should facilitate further development of experimental models incorporating readouts involving lung and organ damage.

From a toxicological perspective, many HDPs cause side effects involving damage to eukaryotic cell membranes. It is therefore notably that EDC34 did not show toxicity at therapeutic doses *in vitro* [32], that the peptides complement boosting activity was only recorded in presence of, and in relation to, bacteria, and that EDC34 was well tolerated in non-infected animals, neither significantly affecting coagulation parameters nor thrombocyte and cytokine levels (**Figure S7**).

In summary, the effects of EDC34, involving blocking of contact activation, and bacterial killing directly or by boosting of complement activation, while maintaining a functional cytokine response (**Figure S8**), represents a potential new approach for boosting bacterial clearance while inhibiting the deleterious effects of excessive pro-coagulative responses during infection with Gram-negative bacteria.

## Materials and Methods

### Ethics statement

The use of human blood was approved by the Ethics Committee at Lund University, Lund, Sweden (Permit Number: 657-2008). Written informed consent was obtained from the donors. The animal experiments were conducted according to national guidelines (Swedish Animal Welfare Act SFS 1988:534) and were approved by the Laboratory Animal Ethics Committee of Malmö/Lund, Sweden (Permit Numbers: M228-10, M252-11)

### Peptides

The peptides EDC34 (EDCKRACAKALKKKKKMPKLRFASRIRKIRKKQF), DAC31 (DACHRACVKGWKKPKRWKIGDFLPRFWKHLS) and DAA14 (DAAQEPTGNNAEIC) were synthesized by Biopeptide Co., San Diego, CA, whereas LL-37 (LLGDFFRKSKEKIGKEFKRIVQRIKDFLRNLVPRTES) was obtained from Innovagen AB, Lund, Sweden. The purity (>95%) of these peptides was confirmed by mass spectral analysis (MALDI-ToF Voyager).

### Microorganisms

The bacterial isolates *E. coli* DH5α, *E. coli* ATCC 25922, *P. aeruginosa* ATCC 27853, *Staphylococcus aureus* ATCC 29213 and *S. pyogenes* AP1 were obtained from the American Type Culture Collection. The *P. aeruginosa* strain PA01 was a generous gift from Dr. B. Iglewski (University of Rochester). *E. coli* O18:K1 was a kind gift from Dr. C. van 't Veer (University of Amsterdam). The clinical isolates *E. coli* 49.1, *E. coli* 47.1 and *P. aeruginosa* 15159 were obtained from the Department of Bacteriology, Lund University Hospital, Sweden.

### Animals

Animals were housed under standard conditions of light and temperature and had free access to standard laboratory chow and water. The animals were purchased from Charles River or the animal facility at Lund University.

### Viable count analysis (VCA)

*E. coli*, *S. aureus* and *S. pyogenes* AP1 strains were grown to mid-exponential phase in Todd-Hewitt (TH) broth. *P. aeruginosa* strains were grown in TH broth overnight. Bacteria were washed and diluted in 10 mM Tris, pH 7.4, containing 0.15 M NaCl, either alone or with 20% normal or heat-inactivated citrate-plasma or 50% citrate blood. Fifty µl bacteria (2×10^6^ cfu/ml) were incubated at 37°C for 2 h with the C-terminal TFPI-2 derived peptide EDC34, LL-37 or DAC31 at the indicated concentrations. Serial dilutions of the incubation mixture were plated on TH agar, followed by incubation at 37°C overnight and cfu determination.

### Hemolysis

Human citrate blood was diluted (1∶1) with PBS prior to addition of 2×10^8^ cfu/ml bacteria. The mixture was incubated with end-over-end rotation for 1 h at 37°C in the presence of peptides (60 and 120 µM). Two percent Triton X-100 (Sigma-Aldrich) served as positive control. The samples were then centrifuged at 800×g for 10 min and the supernatant was transferred to a 96-well microtiter plate. Hemoglobin release was determined by measuring the absorbance at 540 nm and is expressed as % of Triton X-100 induced hemolysis.

### Flow cytometry analysis

Bacteria (1–2×10^9^ cfu) were incubated for 30 min or 1 h at 37°C with human plasma alone or supplemented with TAMRA-labeled EDC34 (at 3 µM). Samples were then prepared for FACS analysis as previously described [52]. For visualization of the complement proteins, rabbit polyclonal antibodies against either LGE27, a C-terminal epitope of human C3a, or rabbit polyclonal antibodies against C1q (both at 1∶100) in combination with a secondary goat anti rabbit IgG FITC-labeled antibody (1∶500, Sigma) were used. Flow cytometry analysis (Becton-Dickinson, Franklin Lakes, NJ) was performed using a FACS Calibur flow cytometry system. The bacterial population was selected by gating with appropriate settings of forward scatter (FSC) and sideward scatter (SSC). Controls without primary antibodies were included. Total positive cells present and fluorescence index (FI) (positive cells present multiplied with mean) are presented in the figures.

### SDS-PAGE and immunoblotting

Human citrate plasma was supplemented with bacteria (1–2×10^9^ cfu) and incubated alone or with EDC34 at 3 µM for 30 min or 1 h at 37°C, centrifuged and supernatants and the bacterial cells were collected. The pull down assay, to extract bound proteins from the bacteria was performed as described previously [52], except two additional wash steps with acetone prior to sample separation by SDS page using 16.5% Tris-tricine gels (C.B.S Scientific) under reducing conditions. Proteins and peptides were transferred to nitrocellulose membranes (Hybond-C), blocked by 3% (w/v) skimmed milk, washed, and incubated with rabbit polyclonal antibodies against the C-terminal part of C3a (LGE27 antibodies) (1∶1000), rabbit polyclonal antibodies to C1q (1∶1000) (Dako) or rabbit polyclonal antibodies to C5b-9 (1∶1000) (Abcam, England). The proteins were detected by using HRP-conjugated secondary antibodies (1∶2000) (Dako) and an enhanced chemiluminesent substrate (LumiGLO) developing system (Upstate cell signaling solutions).

### Clotting assays

All clotting times were analyzed using a coagulometer (Amelung, Lemgo, Germany). For determination of prothrombin time (PT, thromboplastin reagent (Trinity Biotech)) and thrombin clotting time (TCT, Thrombin reagent (Technoclone)), 50 µl of fresh citrate plasma, together with indicated concentrations of EDC34 or DAA14 were pre-warmed for 60 sec at 37°C before clot formation was initiated by adding 50 µl clotting reagent. To record the activated partial thromboplastin time (aPTT), 50 µl of a kaolin-containing solution (Dapttin, Technoclone) was added to the plasma-peptide mix and incubated for 200 sec before clot formation was initiated by adding 50 µl of 30 mM fresh CaCl_2_ solution. To determine the blood clotting time, 50 µl of citrated blood were pre-warmed to 37°C for 60 sec, before 50 µl of 30 mM fresh CaCl_2_ were added to initiate coagulation. Thrombin/antithrombin complexes (TATc) were determined in mouse citrate-plasma by ELISA (USCN Life Sciences Inc.).

### Chromogenic substrate assay

Bacteria were grown in TH broth to mid-exponential phase (OD_620_∼0.5), washed twice in 50 mM Tris/HCl, pH 7.4 and resuspended in 50 mM Tris/HCl, pH 7.4+50 µM ZnCl_2_ to a final concentration of 2×10^9^ cfu/ml. Hundred microliter of bacteria were incubated with 10 µl of EDC34/DAA14 or buffer for 60 sec before the addition of 100 µl human citrate plasma. Samples were incubated for 35 min at 37°C on rotation followed by centrifugation. The bacterial pellets were washed once in 50 mM Tris/HCl, pH 7.4 and resuspended in 100 µl 50 mM Tris/HCl, pH 7.4+50 µM ZnCl_2_ buffer containing 2 mM of the chromogenic substrate S-2302 (Chromogenix). Samples were incubated for 30–60 min at 37°C, centrifuged and the absorbance was measured at 405 nm in the bacterial supernatants. In other experiments 100 µl of Dapttin (Technoclone) were incubated with 10 µl of EDC34/DAA14 for 60 sec prior to the addition of human citrate plasma. Samples were incubated for 3 min at RT, centrifuged and the pellet was washed twice in 50 mM Tris/HCl, pH 7.4 before suspension in 100 µl 50 mM Tris/HCl, pH 7.4+50 µM ZnCl_2_ buffer containing 2 mM of the chromogenic substrate S-2302. After 30 min incubation at RT samples were centrifuged and the absorbance of the supernatant was determined (A405 nm). Samples containing 50 mM Tris/HCl, pH 7.4 instead of citrate plasma served as negative controls.

### Detection of HK degradation

Bacteria and Dapttin samples were prepared as described in the chromogenic substrate assay. Samples were incubated for 15 min at RT, with shaking. Dapttin samples were centrifuged at 10.000 rpm for 2 min and supernatants were stored at −20°C. Bacterial samples were washed twice in 50 mM Tris/HCl, pH 7.4 and resuspended in 55 µl 50 mM Tris/HCl, pH 7.4+50 µM ZnCl_2_ buffer followed by 15 min incubation at RT before centrifugation at 10.000 rpm for 2 min and storage of supernatants at −20°C. Samples were separated by SDS page and analyzed by western blot using antibodies against HK and its degradation products as previously described [53].

### Bradykinin assay

Human citrate plasma was incubated with 50 µM of EDC34 or DAA14 for 2 min at RT before the addition of Dapttin and incubation for 2 min at 37°C. Samples were kept on ice, diluted with deproteinising buffer (1∶5), centrifuged (at 4°C) and the pellet was mixed with equal amounts of assay buffer. Samples with H_2_O instead of peptide served as positive controls. All buffers were provided together with the ELISA kit used to quantify the released bradykinin according to manufactures instructions (Markit-M-Bradykinin Kit; DS Pharma Biomedical co.Ltd).

### LPS animal model

Male C57BL/6 mice (8 weeks) were i. p. injected with 5 mg/kg of *E. coli* O111:B4 LPS (Sigma). Thirty minutes after LPS challenge mice were treated with 0.5 mg EDC34 (i. p.). For analysis of cytokines and coagulation parameters, mice were sacrificed 4 and 8 h post-LPS injection and the blood was collected by cardiac puncture.

### *E. coli* infection model

*E. coli* DH5-α bacteria were grown to mid-exponential phase (OD_620_∼0.5), harvested, washed in PBS and diluted in the same buffer to 2×10^9^ cfu/ml. Hundred fifty microliter of the bacterial suspension was injected intraperitoneally (i. p.) into male BALB/c mice immediately followed by 0.5 mg EDC34 or PBS. Mice were sacrificed 0.5, 2, 4 and 8 h post-infection to evaluate cfu, cytokines, coagulation parameters, blood counts and histology of the lungs. In another set of experiments mice were treated with 0.5 mg EDC34 injected i. p., immediately, or after 1 h, or subcutaneously 1 h post-infection. The animal status and weight was followed daily for up to 7 days. Mice sacrificed before day 7, according to predefined endpoint criteria, were counted as non-survivors. In another experiment, male BALB/c mice were depleted of complement factors by i. p. injection of 4.8 U of cobra venom factor (CVF) (Quidel) [39]. After 16 h mice were infected with *E. coli* DH5-α (i. p.) and immediately treated with 0.5 mg EDC34 or PBS. Mice were sacrificed 6 h post-infection and cfu were evaluated in spleen and liver, respectively.

### *P. aeruginosa* infection model

*P. aeruginosa* 15159 or *P. aeruginosa* PA01 bacteria were grown to mid-exponential phase (OD_620_∼0.5), harvested, washed in PBS, diluted in the same buffer to 2×10^9^ cfu/ml, and kept on ice until injection. Hundred microliter of the bacterial suspension was injected (i. p.) into male C57BL/6 mice. EDC34 (0.5 mg) or buffer alone was administered i. p. immediately after bacterial injection, or s. c. either as one dose after 1 h, or two doses at 1 h and 7 h. In another experiment, mice were treated twice s. c with a combination of 300 mg/kg ceftazidime and 0.5 mg of EDC34, 1.5 h and 4.5 h after bacterial infection. Data from three independent experiments were pooled. The survival data were obtained by following the animals daily up to 7 days monitoring status and weight. Mice reaching the predefined endpoint-criteria were sacrificed and counted as non-survivors.

### Histochemistry

For histological evaluation of lungs derived from the *in vivo P. aeruginosa* infection model, tissues were collected at indicated time points, fixed in 4% formaldehyde for 24 h, embedded in paraffin, sectioned and stained with hematoxylin and eosin. Assessment of differences in alveolar space, cell infiltration, thickness of alveolar septa (cell wall thickness) and thrombi (Figure S6) was performed by scoring of at least five view fields per section by three blinded independent observers (score 1–4; where 1 indicates no change, 2 minor, 3 medium, and 4 significant change).

### Electron microscopy

For transmission electron microscopy analysis of the presence of TFPI-2 fragments *in vivo*, fibrin slough from a patient with a chronic venous ulcer (CWS) was fixed and processed as previously described [52]. For immunostaining [52], rabbit polyclonal antibodies against the C-terminal of TFPI-2 (CAKALKKKKKMPKLRFASRIRKIRKKQF) alone, or in combination with rabbit polyclonal antibodies against the C-terminal part of C3a (LGE27 antibodies) (1 µg/ml) (Innovagen AB) were utilized. Controls without primary antibodies were also included. For simultaneous detection of TFPI-2 and C3a, 1 µg/ml EM rabbit anti-goat IgG 20 nm Au (BBI) and 1 µg/ml EM goat anti-rabbit IgG 10 nm Au (BBI) were used. All samples were examined with a Jeol JEM 1230 electron microscope operated at 80 kV accelerating voltage. Images were recorded with a Gatan Multiscan 791 charge-coupled device camera. For scanning electron microscopy, lungs were collected at 12 h after injection of bacteria and fixed in 2.5% (v/v) glutaraldehyde in 0.15 M sodium cacodylate buffer, pH 7.4, overnight at room temperature and further treated as described previously [31]. Specimens were examined in a JEOL JSM-350 scanning electron microscope. To quantify pulmonary lesions, lung samples from 30 different fields covering an entire lung section were made, and the percentage of fibrin deposits and fields exhibiting hemorrhage were determined.

### Cytokine assay

The cytokines IL-6, IL-10, MCP-1, IFN-γ, and TNF-α were measured in mouse plasma using the Cytometric bead array; Mouse Inflammation Kit (Becton Dickinson AB) according to the manufacturer's instructions.

### Blood cell counts

The number of platelets in mouse blood anti-coagulated with EDTA was determined using the VetScan HM5 (TrioLab).

### Statistical analysis

Values are shown as mean with SEM. For statistical evaluation of two experimental groups the Mann-Whitney U-test was used and for comparison of survival curves the log-rank test. To compare more than two groups One-Way or Two-Way ANOVA with Bonferoni post-test were used. Viable count data are presented as mean with SD. All statistical evaluations were performed using the GraphPad Prism software 5.0. with *p-<0.05, **<0.01 and ***p<0.001 and ns = not significant.

## Supporting Information

Figure S1 **Antimicrobial activities of EDC34 and LL-37.** (**A–B**) Antibacterial effects of EDC34 and LL-37 against (**A**) *E. coli* or (**B**) *P. aeruginosa* strains in viable count assays performed in 10 mM Tris, 0.15 M NaCl, pH 7.4 (Buffer), or in 10 mM Tris, 0.15 M NaCl, pH 7.4, containing 20% human citrate plasma (CP), or heat-inactivated human citrate plasma (HCP). (**C–D**) Antimicrobial activity in human blood. Bacteria were incubated with EDC34 or LL-37 in 50% human citrate blood (diluted in PBS) and the number of cfu was determined. (n = 3, mean±SD presented).
(TIF)

Figure S2 **Kinetics of bacterial killing by EDC34 and LL-37.** (**A–B**) Viable count assays of indicated *E. coli* (**A**) or *Pseudomonas* (**B**) isolates were subjected to EDC34 or LL-37 at 3 µM in 10 mM Tris, 0.15 M NaCl, pH 7.4, containing 20% human citrate plasma (CP). Mean with SD is shown (n = 3).
(TIF)

Figure S3 **EDC34 enhances the binding of complement proteins to bacteria.** Examples of flow cytometry histograms of C1q/C3a binding to *E. coli* and *P. aeruginosa* in citrate plasma in absence (Control) or presence of EDC34.
(TIF)

Figure S4 **EDC34 does not inhibit inflammatory responses** ***in vitro*** **.** Mouse macrophages were stimulated with 10 ng/ml *E. coli* LPS with or without increasing concentrations EDC34 or GKY25. Nitric oxide release was determined by using the Griess reaction in cell supernatants after 20 h. Data are shown as mean±SEM (n = 3).
(TIF)

Figure S5 **Role of complement on EDC34 mediated killing** ***in vivo*** **.** (**A–B**) Male Balb/c mice were infected with *E. coli* DH5-α bacteria (i. p.) followed by immediate treatment with 0.5 mg EDC34 (i. p.). C3a was determined in (**A**) peritoneal fluid (Non-infected mice (Control); n = 8, EDC34; n = 6, *E. coli* infection (*E. coli*); n = 13, treatment with EDC34; n = 13) and (**B**) plasma (Non-infected mice (Control); n = 10, EDC34; n = 6, *E. coli* infection (*E. coli*); n = 15, treatment with EDC34; n = 15; Two-Way ANOVA Bonferroni's Multiple Comparison Test) at the indicated time points. (**C**) In a separate experiment, a group of Balb/c mice were pre-treated with cobra venom factor (CVF) 16 h prior to infection with *E. coli* DH5-α bacteria (*E. coli*), and treatment with EDC34 as above. After 2 h post-infection cfu were determined in spleen and liver (*E. coli*; n = 4, EDC34; n = 5, CVF+ *E. coli*; n = 6, and CVF+*E. coli*+EDC34; n = 6).
(TIF)

Figure S6 **Histology score.** C57BL/6 mice were infected (i. p.) with *P. aeruginosa* bacteria (2×10^9^ cfu/ml) and s. c. treated with either the antibiotic ceftazidime (AB) (300 mg/kg), EDC34 (0.5 mg) or a combination of both 1.5 h and 4.5 h post-infection. Mice were sacrificed 10 h post-infection. Histology scores of hematoxylin-eosin stained lung sections, according to the indicated criteria are shown. Values are presented as mean ± SEM (Control; n = 5, *P. aeruginosa* infection, treatment of infection with EDC34, ceftazidime (AB) or a combination of both (AB+EDC34); n = 10 for all groups; Two-Way ANOVA Bonferroni's Multiple Comparison Test).
(TIF)

Figure S7 ***In vivo*** **effects of EDC34 alone.** (**A–B**) 1 mg of EDC34 was administered i. p. two times (0 and 1.5 h) into healthy male C57BL/6 mice. Mice were sacrificed 12 h after the first injection and (**A**) clotting times, (**B**) cytokines were evaluated. Mean±SEM is presented (Control; n = 3, EDC34; n = 6, nonparametric Mann-Whitney t-test, ns; not significant).
(TIF)

Figure S8 **Proposed actions of the peptide EDC34 during Gram-negative bacterial infection.**
(TIF)

Methods S1 **C3a assay.**
(DOCX)

Methods S2 **Nitrite assay.**
(DOCX)

Table S1 **C-terminal sequences of human and mouse TFPI-2.**
(DOCX)
