# Supplementary figures and images for: The TFPI-2 Derived Peptide EDC34 Improves Outcome of Gram-Negative Sepsis
Source: PLoS Pathog. 2013 Dec 5;9(12):e1003803. doi: 10.1371/journal.ppat.1003803 (PMC3855554; doi:10.1371/journal.ppat.1003803)

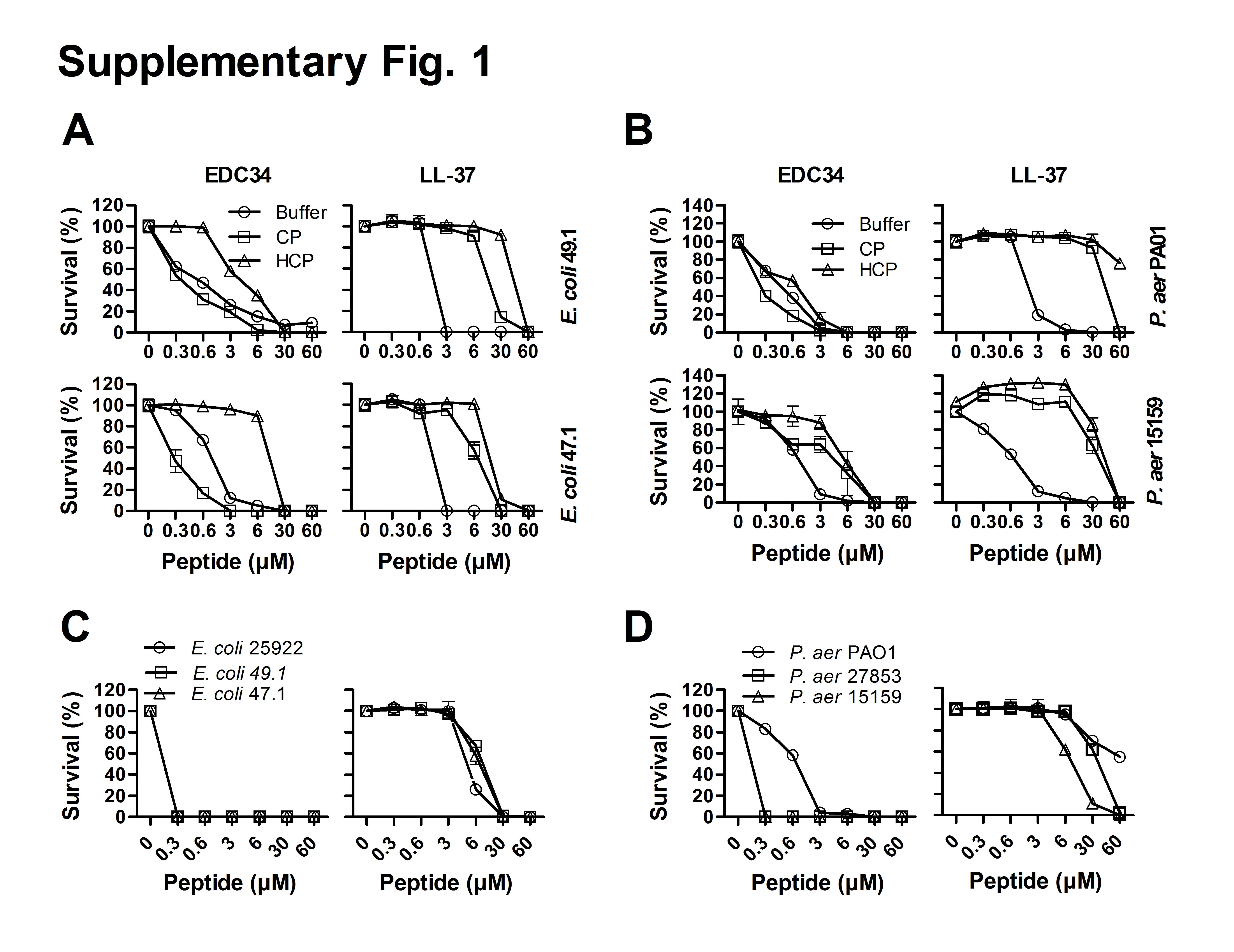

Supplement: Figure S1 — Antimicrobial activities of EDC34 and LL-37. (A–B) Antibacterial effects of EDC34 and LL-37 against (A) E. coli or (B) P. aeruginosa strains in viable count assays performed in 10 mM Tris, 0.15 M NaCl, pH 7.4 (Buffer), or in 10 mM Tris, 0.15 M NaCl, pH 7.4, containing 20% human citrate plasma (CP), or heat-inactivated human citrate plasma (HCP). (C–D) Antimicrobial activity in human blood. Bacteria were incubated with EDC34 or LL-37 in 50% human citrate blood (diluted in PBS) and the number of cfu was determined. (n = 3, mean±SD presented). (TIF) [file ppat.1003803.s001.tif]

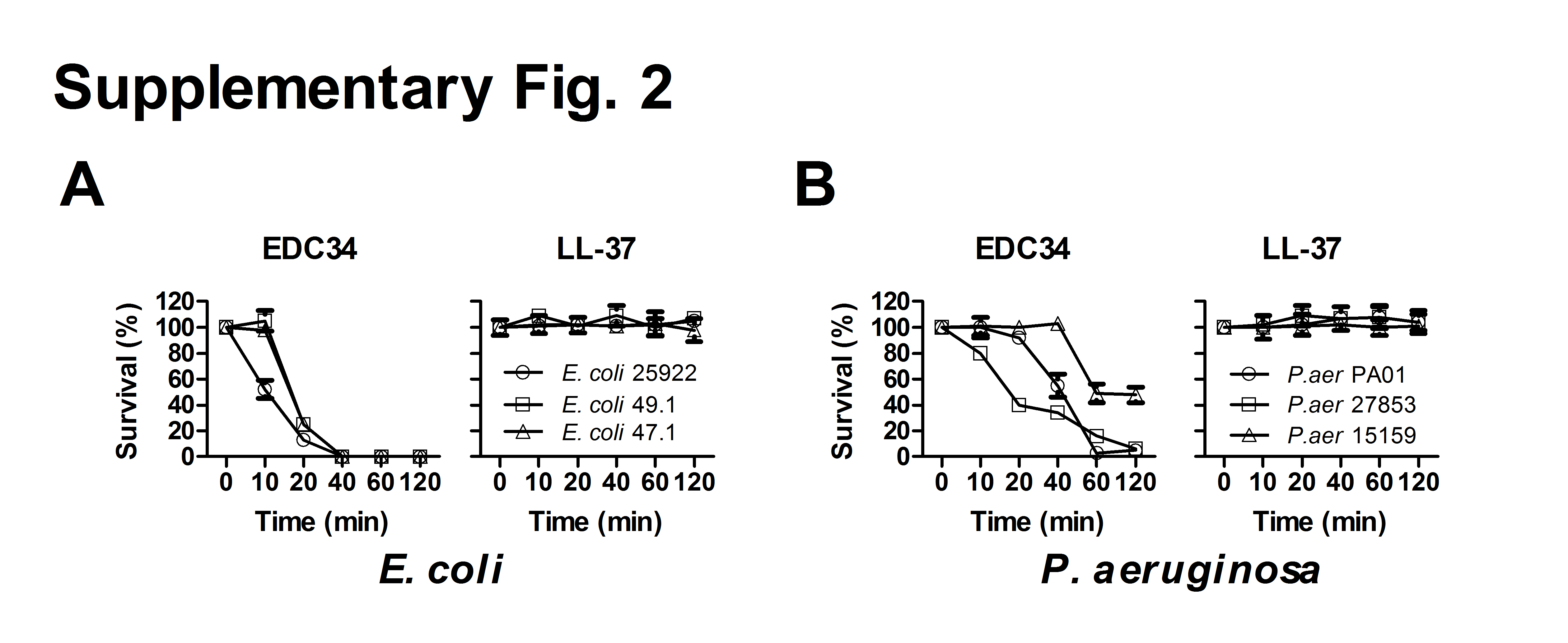

Supplement: Figure S2 — Kinetics of bacterial killing by EDC34 and LL-37. (A–B) Viable count assays of indicated E. coli (A) or Pseudomonas (B) isolates were subjected to EDC34 or LL-37 at 3 µM in 10 mM Tris, 0.15 M NaCl, pH 7.4, containing 20% human citrate plasma (CP). Mean with SD is shown (n = 3). (TIF) [file ppat.1003803.s002.tif]

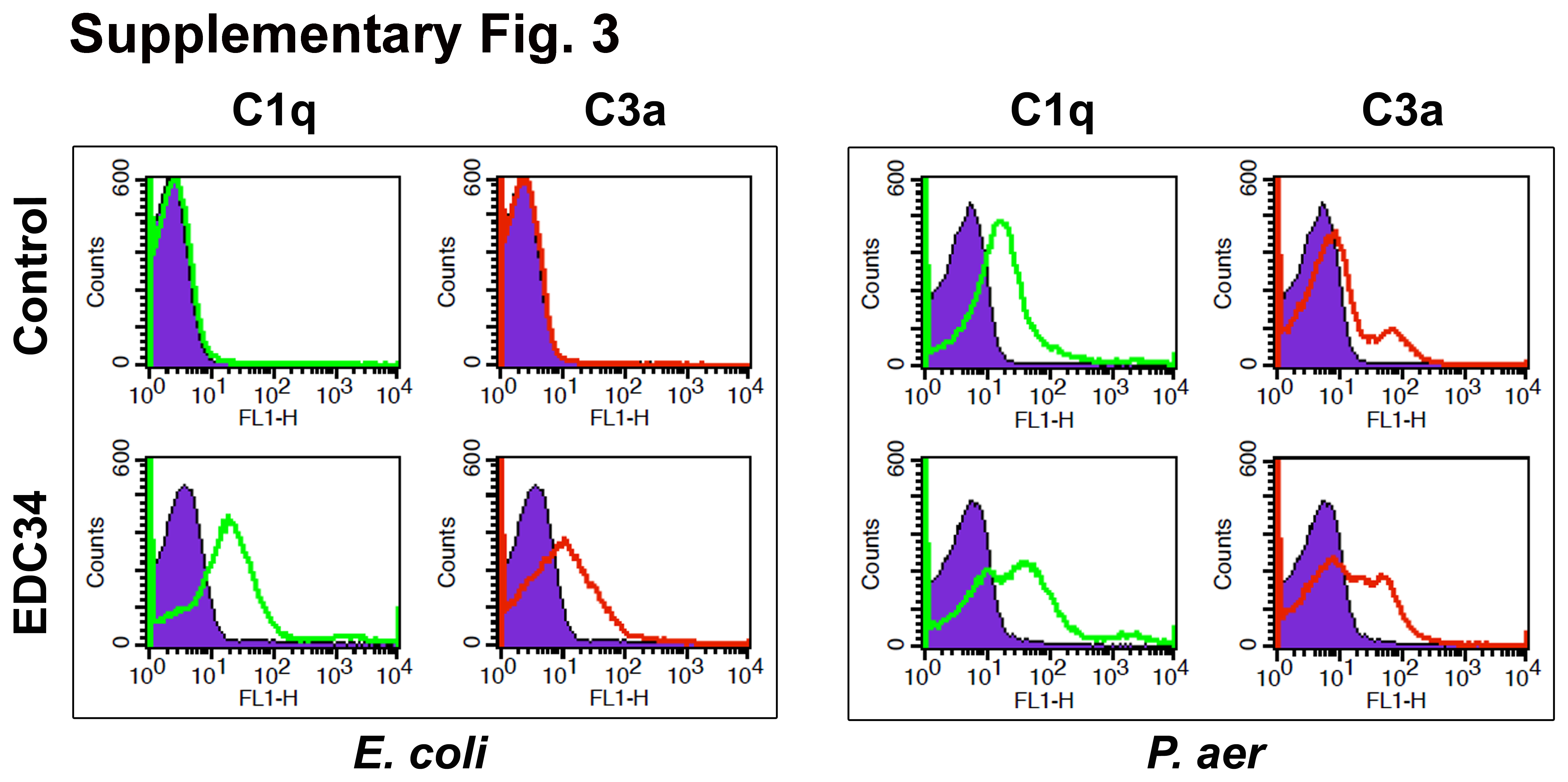

Supplement: Figure S3 — EDC34 enhances the binding of complement proteins to bacteria. Examples of flow cytometry histograms of C1q/C3a binding to E. coli and P. aeruginosa in citrate plasma in absence (Control) or presence of EDC34. (TIF) [file ppat.1003803.s003.tif]

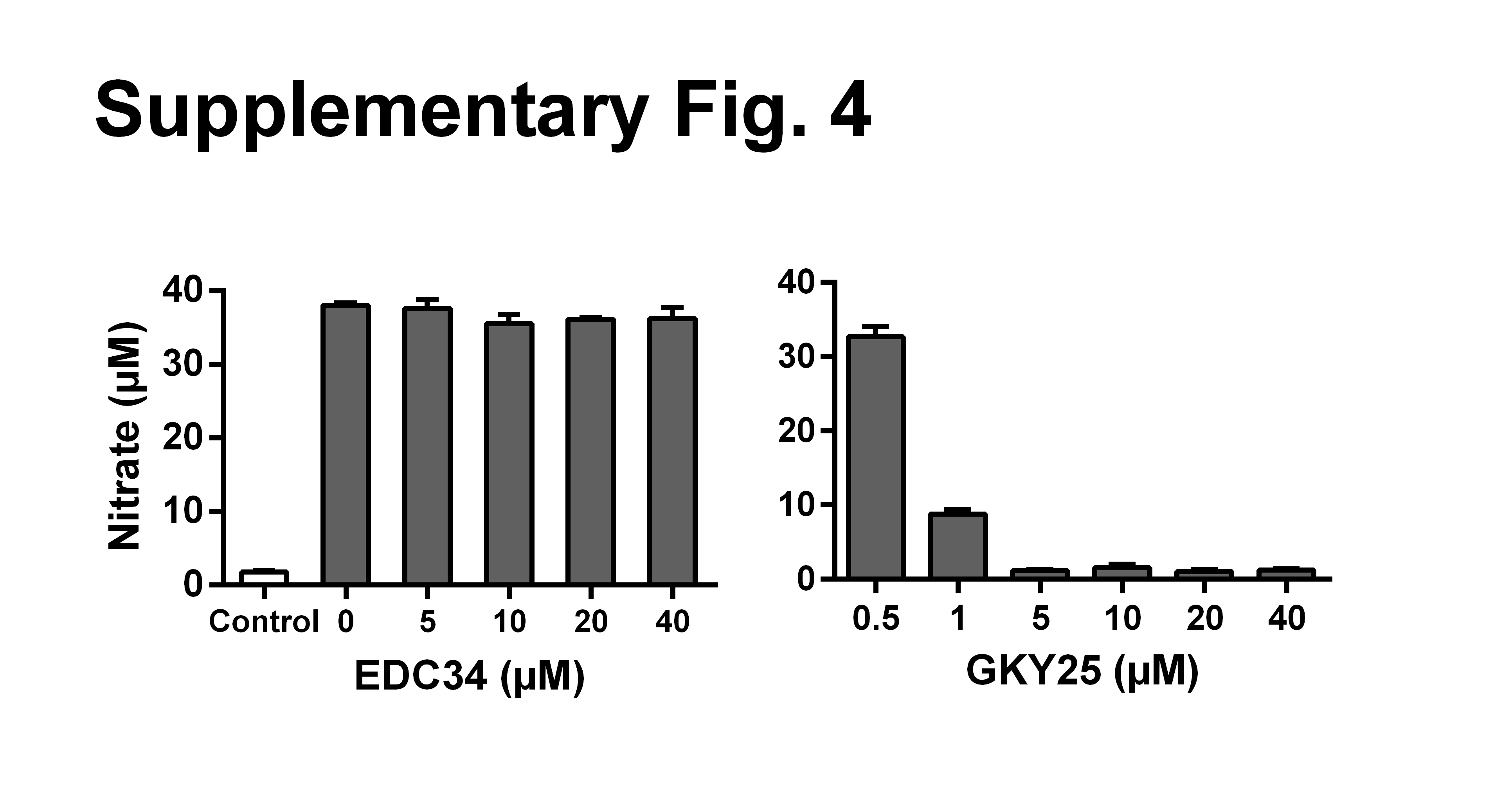

Supplement: Figure S4 — EDC34 does not inhibit inflammatory responses in vitro . Mouse macrophages were stimulated with 10 ng/ml E. coli LPS with or without increasing concentrations EDC34 or GKY25. Nitric oxide release was determined by using the Griess reaction in cell supernatants after 20 h. Data are shown as mean±SEM (n = 3). (TIF) [file ppat.1003803.s004.tif]

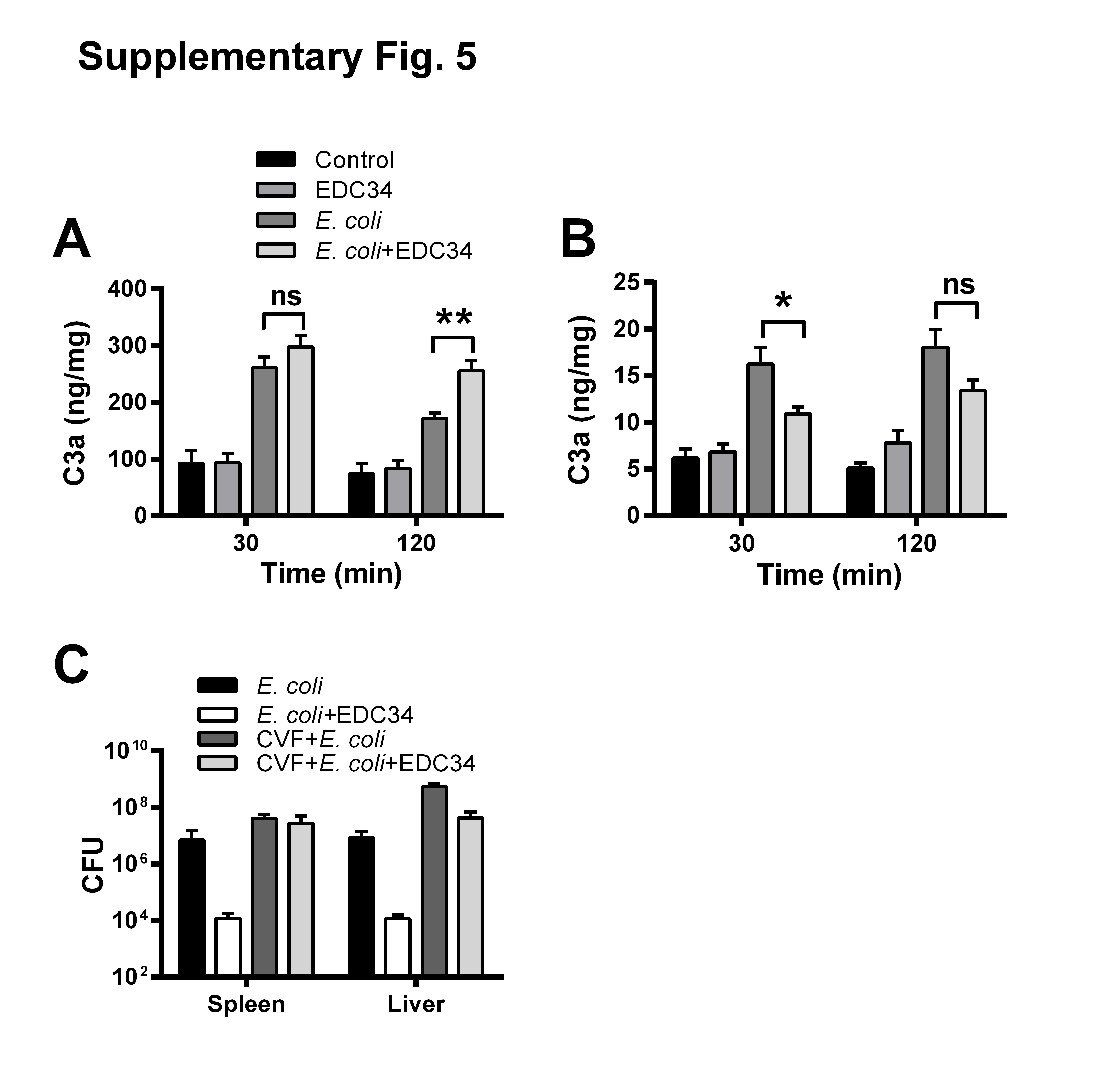

Supplement: Figure S5 — Role of complement on EDC34 mediated killing in vivo . (A–B) Male Balb/c mice were infected with E. coli DH5-α bacteria (i. p.) followed by immediate treatment with 0.5 mg EDC34 (i. p.). C3a was determined in (A) peritoneal fluid (Non-infected mice (Control); n = 8, EDC34; n = 6, E. coli infection (E. coli); n = 13, treatment with EDC34; n = 13) and (B) plasma (Non-infected mice (Control); n = 10, EDC34; n = 6, E. coli infection (E. coli); n = 15, treatment with EDC34; n = 15; Two-Way ANOVA Bonferroni's Multiple Comparison Test) at the indicated time points. (C) In a separate experiment, a group of Balb/c mice were pre-treated with cobra venom factor (CVF) 16 h prior to infection with E. coli DH5-α bacteria (E. coli), and treatment with EDC34 as above. After 2 h post-infection cfu were determined in spleen and liver (E. coli; n = 4, EDC34; n = 5, CVF+ E. coli; n = 6, and CVF+E. coli+EDC34; n = 6). (TIF) [file ppat.1003803.s005.tif]

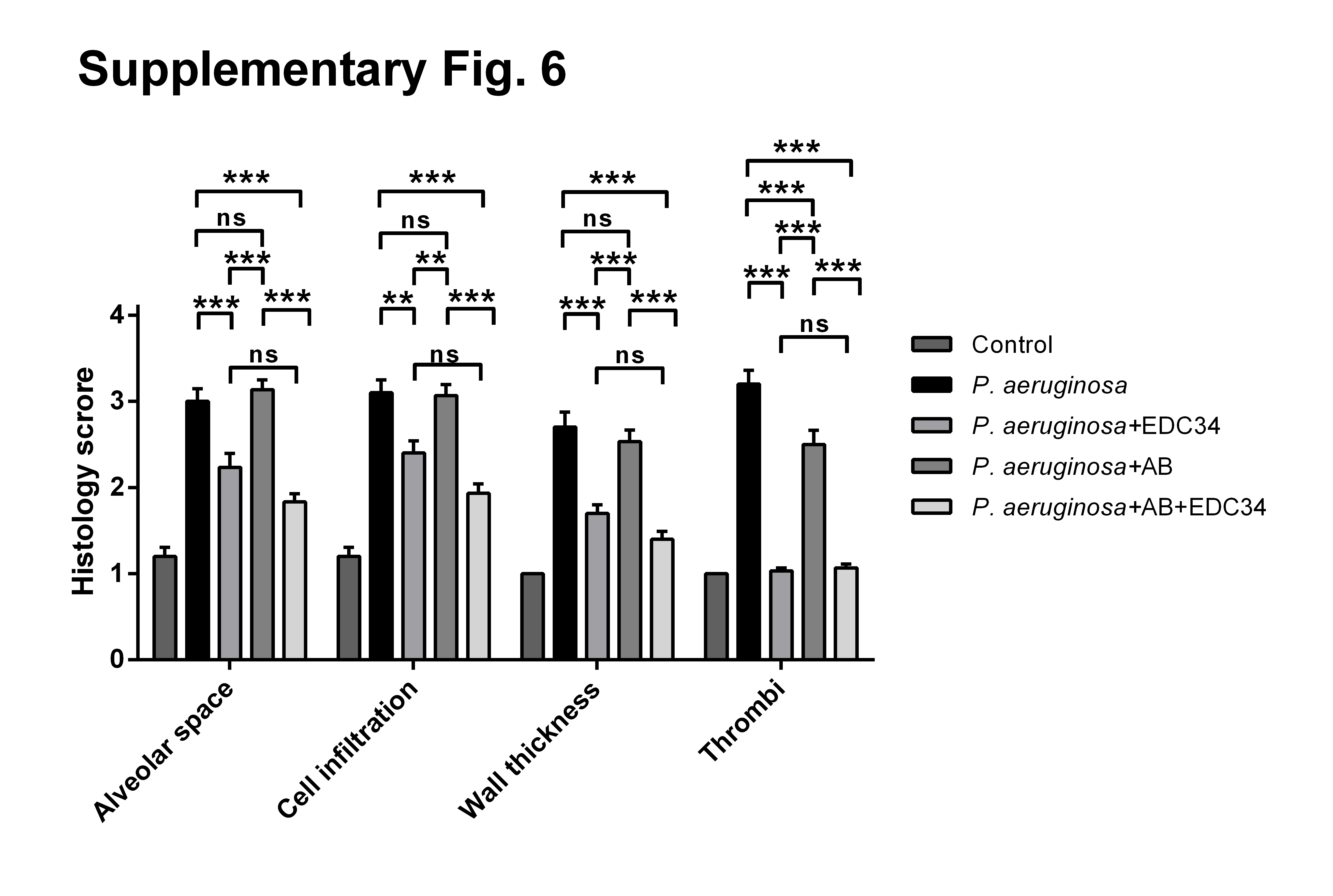

Supplement: Figure S6 — Histology score. C57BL/6 mice were infected (i. p.) with P. aeruginosa bacteria (2×10^9^ cfu/ml) and s. c. treated with either the antibiotic ceftazidime (AB) (300 mg/kg), EDC34 (0.5 mg) or a combination of both 1.5 h and 4.5 h post-infection. Mice were sacrificed 10 h post-infection. Histology scores of hematoxylin-eosin stained lung sections, according to the indicated criteria are shown. Values are presented as mean ± SEM (Control; n = 5, P. aeruginosa infection, treatment of infection with EDC34, ceftazidime (AB) or a combination of both (AB+EDC34); n = 10 for all groups; Two-Way ANOVA Bonferroni's Multiple Comparison Test). (TIF) [file ppat.1003803.s006.tif]

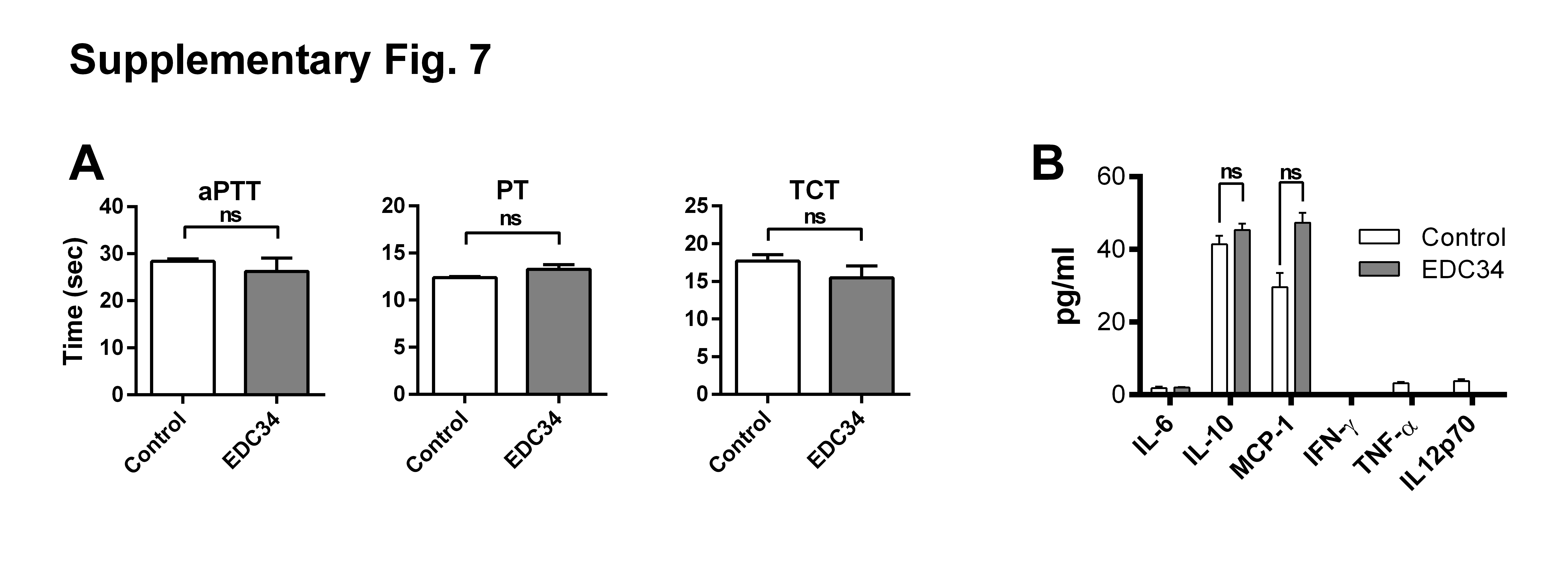

Supplement: Figure S7 — In vivo effects of EDC34 alone. (A–B) 1 mg of EDC34 was administered i. p. two times (0 and 1.5 h) into healthy male C57BL/6 mice. Mice were sacrificed 12 h after the first injection and (A) clotting times, (B) cytokines were evaluated. Mean±SEM is presented (Control; n = 3, EDC34; n = 6, nonparametric Mann-Whitney t-test, ns; not significant). (TIF) [file ppat.1003803.s007.tif]

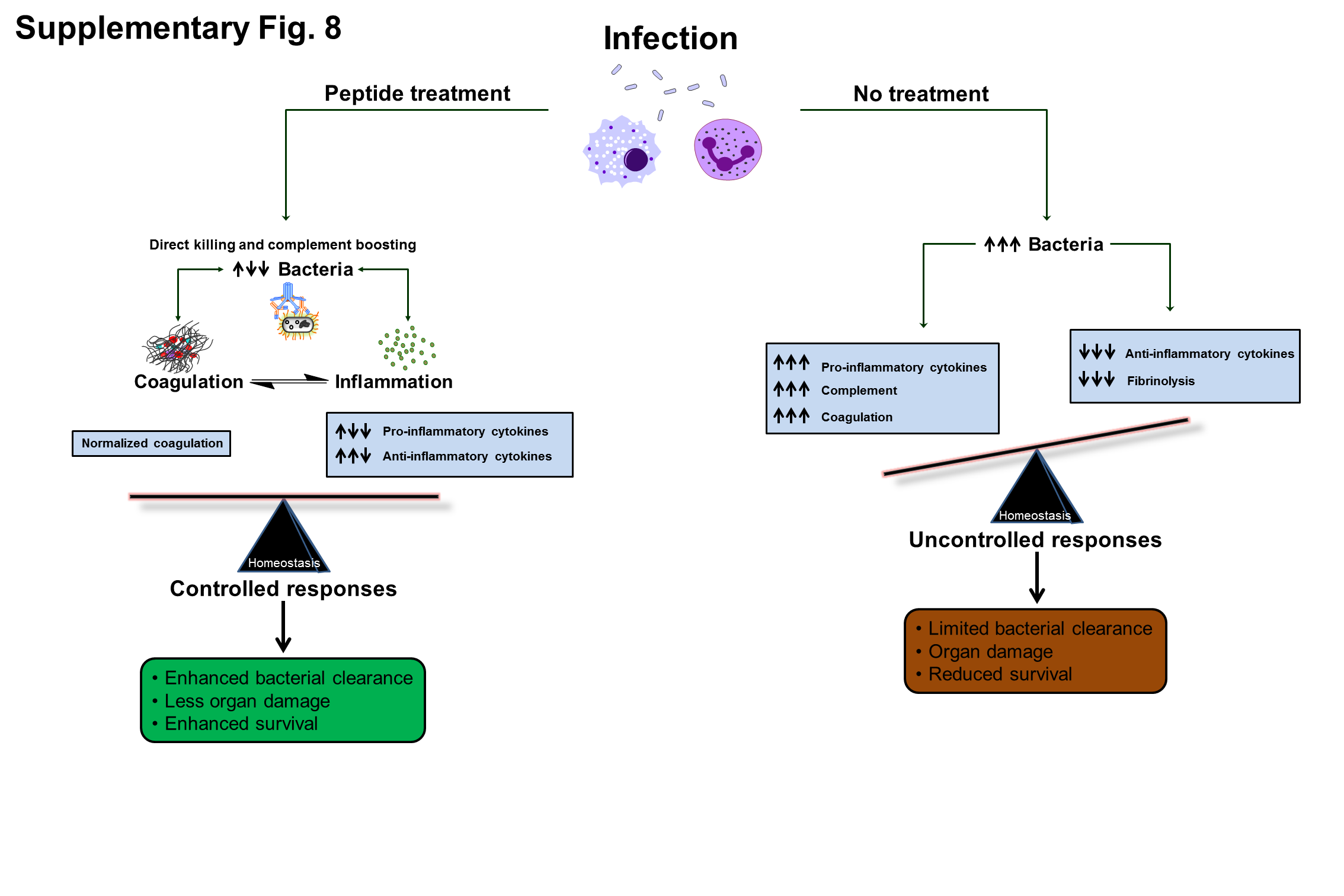

Supplement: Figure S8 — Proposed actions of the peptide EDC34 during Gram-negative bacterial infection. (TIF) [file ppat.1003803.s008.tif]
