# Supplementary material for: The TFPI-2 Derived Peptide EDC34 Improves Outcome of Gram-Negative Sepsis
Source: PLoS Pathog. 2013 Dec 5;9(12):e1003803. doi: 10.1371/journal.ppat.1003803 (PMC3855554; doi:10.1371/journal.ppat.1003803)
Supplement: Methods S1 — C3a assay. (DOCX) [file ppat.1003803.s009.docx]

**Methods S1**

**C3a assay.** C3a was measured in plasma or peritoneal wash samples of Balb/c mice using a sandwich enzyme-linked immunosorbent assay (ELISA). In brief, 96-well plates (Nunc, Maxisorb) were coated with 2 μg/ml of a rat-anti mouse C3a antibody (BD Bioscience) in 0.2 M sodium phosphate buffer. Detection of C3a was done by using 1 μg/ml of a biotin labeled rat-anti mouse C3a antibody (BD Bioscience) and Avidin–horse radish peroxidase (BD Bioscience). C3a values were normalized to the total protein content of the sample, which was determined by using the PierceTM BCA protein assay kit (Thermo Scientific). Measurement was done according to the manufacturer’s instructions.
