# Supplementary material for: The TFPI-2 Derived Peptide EDC34 Improves Outcome of Gram-Negative Sepsis
Source: PLoS Pathog. 2013 Dec 5;9(12):e1003803. doi: 10.1371/journal.ppat.1003803 (PMC3855554; doi:10.1371/journal.ppat.1003803)
Supplement: Methods S2 — Nitrite assay. (DOCX) [file ppat.1003803.s010.docx]

**Methods S2**

**Nitrite assay**. RAW 264.7 macrophages (3.5×10^6^ cells/ml) (ATCC, Rockville, MD) in phenol red-free Dulbecco´s modified Eagle medium (DMEM; PAA-Laboratories) supplemented with 10% (v/v) heat-inactivated fetal bovine serum (FBS) (Invitrogen) and 1% (v/v) Antibiotic-Antimycotic solution (AAS) (Invitrogen) were stimulated with 10 ng/ml *E. coli* LPS (0111:B4 (Sigma-Aldrich, approximate 500.000 endotoxin units/mg) together with or without various concentrations of EDC34 or GKY25. The level of nitrite oxide (NO) presented as Nitrite (µM) in culture supernatants was determined after 20 h as described previously [[1](#_ENREF_1)].

1. Kalle M, Papareddy P, Kasetty G, Morgelin M, van der Plas MJ, et al. (2012) Host Defense Peptides of Thrombin Modulate Inflammation and Coagulation in Endotoxin-Mediated Shock and Pseudomonas aeruginosa Sepsis. PLoS One 7: e51313.
