# Supplementary material for: The TFPI-2 Derived Peptide EDC34 Improves Outcome of Gram-Negative Sepsis
Source: PLoS Pathog. 2013 Dec 5;9(12):e1003803. doi: 10.1371/journal.ppat.1003803 (PMC3855554; doi:10.1371/journal.ppat.1003803)
Supplement: Table S1 — C-terminal sequences of human and mouse TFPI-2. (DOCX) [file ppat.1003803.s011.docx]

**Table-1**

| **Species** | **Sequence** | **Charge** |
| --- | --- | --- |
| Human | **EDCKRACAKALKKKKKMPKLRFASRIRKIRKKQF** | +14 |
| Mouse | **DACHRACVKGWKKPKRWKIGDFLPRFWKHLS** | +7 |
